# Physiological classification of Parkinson’s disease severity using multimodal speech biomarkers with a hybrid CNN-Mamba framework

**DOI:** 10.3389/fphys.2026.1806415

**Published:** 2026-05-18

**Authors:** Taisheng Zeng, Yuguang Ye, Yunyi Zeng, Jianshe Shi, Yifeng Huang, Bijiao Ding, Kavimbi Chipusu, Jianlong Huang

**Affiliations:** 1 School of Mathematics and Computer Science, Quanzhou Normal University, Quanzhou, China; 2 Fujian Provincial Key Laboratory of Data-Intensive Computing, Quanzhou Normal University, Quanzhou, China; 3 Key Laboratory of Intelligent Computing and Information Processing (Quanzhou Normal University), Fujian Province University, Quanzhou, China; 4 Department of Mathematics and Information Technology, The Education University of Hong Kong, Hong Kong, Hong Kong SAR, China; 5 Department of Diagnostic Radiology, Huaqiao University Affiliated Strait Hospital, Quanzhou, Fujian, China; 6 Department of Mechanical Engineering, Division of Biomedical Engineering, University of Saskatchewan, Saskatoon, SK, Canada

**Keywords:** CNN-Mamba, cross-lingual generalization, interpretable deep learning, multi-class severity classification, non-invasive diagnosis, Parkinson’s disease, selective state space model, speech biomarkers

## Abstract

**Introduction:**

Hypokinetic dysarthria in Parkinson’s disease provides an accessible non-invasive biomarker, but multi-class severity grading remains difficult because of overlapping acoustic patterns and limited long-range temporal modeling in existing approaches.

**Methods:**

We developed a hybrid CNN-Mamba framework using multimodal speech features transformed into 2D representations. The model was trained and validated on speaker-disjoint PC-GITA Spanish data and tested on an independent Mandarin clinical cohort, with additional external evaluation on a public Parkinsonian speech corpus. Speaker-level results were obtained by aggregating segment predictions within each subject.

**Results:**

Segment-level accuracy reached 97.8% on PC-GITA and 95.4% on the Mandarin cohort. Speaker-level accuracy reached 94.0% and 91.2% using majority voting, improving to 94.8% and 91.9% with mean-probability aggregation. SHAP analysis supported physiological interpretability, and ablation studies showed advantages over CNN-BiLSTM, Transformer, and SVM baselines.

**Discussion:**

The proposed CNN-Mamba framework provides an interpretable, computationally efficient, and non-invasive approach for Parkinson’s disease severity assessment and remote monitoring, with promising cross-lingual transfer under structured clinical speech tasks.

## 1 Introduction

Parkinson’s disease (PD) is one of the fastest-growing neurodegenerative disorders worldwide, primarily caused by the progressive loss of dopaminergic neurons in the substantia nigra pars compacta. This results in debilitating motor symptoms such as resting tremor, bradykinesia, rigidity, and postural instability, as well as a wide range of non-motor manifestations, including depression, autonomic dysfunction, and cognitive decline (Armstrong and Okun, 2020; Bloem et al., 2021). These impairments significantly reduce patients’ quality of life and independence, placing a substantial socioeconomic burden on healthcare systems (Dorsey et al., 2018).

Recent projections from the Global Burden of Disease Study 2021 estimate that global PD prevalence will surge to 25.2 million cases (95% UI: 21.7–30.1 million) by 2050, representing a staggering 112% increase (95% UI: 71–152%) from 2021 levels. This increase is largely attributed to population ageing (approximately 89%), with additional contributions from population growth (~20%) and slight changes in prevalence (~3%) (Su et al., 2025). The all-age prevalence is expected to reach 267 per 100,000 (a 76% rise), while age-standardized prevalence will increase by 55%, reaching 216 per 100,000 (Su et al., 2025). The highest burden is anticipated in middle Socio-Demographic Index regions (with up to 144% relative increase) and East Asia (projected to have 10.9 million cases by mid-century) (Cao et al., 2025; Su et al., 2025). This dramatic growth underscores the urgent need for accessible, scalable, and early diagnostic tools, particularly in resource-limited and aging societies where conventional methods—such as subjective clinical assessments (e.g., UPDRS) and expensive neuroimaging (e.g., dopamine transporter scans) are often delayed, costly, and inaccessible (Sapmaz Atalar et al., 2023). At the same time, neuroimaging-based computational studies continue to provide important mechanistic and functional insight into brain-level abnormalities and treatment-related effects in neurological disorders, highlighting the complementary role of data-driven analytics across clinical modalities (Wong et al., 2023c).

Among non-invasive biomarkers, speech impairments collectively referred to as hypokinetic dysarthria—are among the earliest, most prevalent, and digitally quantifiable signs of PD (Karki et al., 2023; Rusz et al., 2024). Affecting up to 90% of patients, dysarthria often appears in prodromal or very early stages, sometimes even years before prominent motor symptoms (Krohova et al., 2025). It is characterized by reduced vocal loudness, monotone prosody, imprecise articulation, elevated jitter and shimmer, breathiness, and disrupted rhythm. These symptoms are direct consequences of bradykinesia and rigidity, which impact respiratory, laryngeal, and articulatory control (van Gelderen and Tejedor-García, 2023; Rusz et al., 2024). The acoustic changes associated with dysarthria produce rich, easily capturable signals via smartphones or simple recorders, allowing for low-cost, remote, and scalable screening with minimal patient effort (Alaskar et al., 2025; Xu et al., 2025). Automated speech analysis has also shown value in early untreated Parkinson’s disease and has been associated with dopaminergic transporter imaging abnormalities (Rusz et al., 2022).

Artificial intelligence, particularly deep learning, has revolutionized speech-based PD detection by moving from hand-crafted features to end-to-end models that learn complex spatio-temporal patterns directly from audio (Gu and Dao, 2023). However, most studies have focused on binary classification (PD vs. healthy), achieving high accuracy in controlled settings. Multi-class speech classification using ordinal severity levels plus phenotype-aware subgrouping (e.g., Normal, Mild, Moderate, Severe, Tremor-dominant) remains challenging due to subtle inter-class acoustic overlaps, subjective perceptual labeling, and limitations in modeling long-range temporal dependencies (e.g., progressive monotonicity or tremor modulation over extended utterances) (Shams et al., 2024; Yadav and Tan, 2024; Zhang et al., 2025). However, tremor-related modulation is treated here as an exploratory phenotype-associated signal rather than an established standalone speech-severity marker. Traditional hybrid models, such as CNN-RNN/LSTM, suffer from sequential bottlenecks and gradient issues, while Transformers incur quadratic computational costs for long sequences, which hinders their efficiency in real-world, variable-length speech data (Erol et al., 2024).

To address these gaps, we introduce a novel hybrid CNN-Mamba framework, marking, to the best of our knowledge, the first application of selective state space modeling (Mamba) for multi-class PD severity classification from speech. Mamba’s input-dependent discretization and linear-time selective scanning enable efficient capture of extended dysarthria patterns without the quadratic scaling of attention or the recurrence of RNNs (Misra, 2019; Goel et al., 2022). By combining CNN for local spectro-temporal feature extraction from multimodal acoustic representations (MFCCs, jitter, shimmer, wavelet entropy, and glottal parameters), the model delivers superior performance, generalization, and computational efficiency. More broadly, hybrid deep learning architectures that combine convolutional feature extraction with advanced sequence or transformer-inspired modeling have shown strong performance across biomedical image analysis and cross-modality clinical decision tasks, supporting the use of similarly integrated designs in clinically oriented pattern-recognition problems (Ding et al., 2022; Xie et al., 2022; Zhao et al., 2022; Ayoub et al., 2023; Wong et al., 2023b; Wong et al., 2023a).

**Figure 1:**
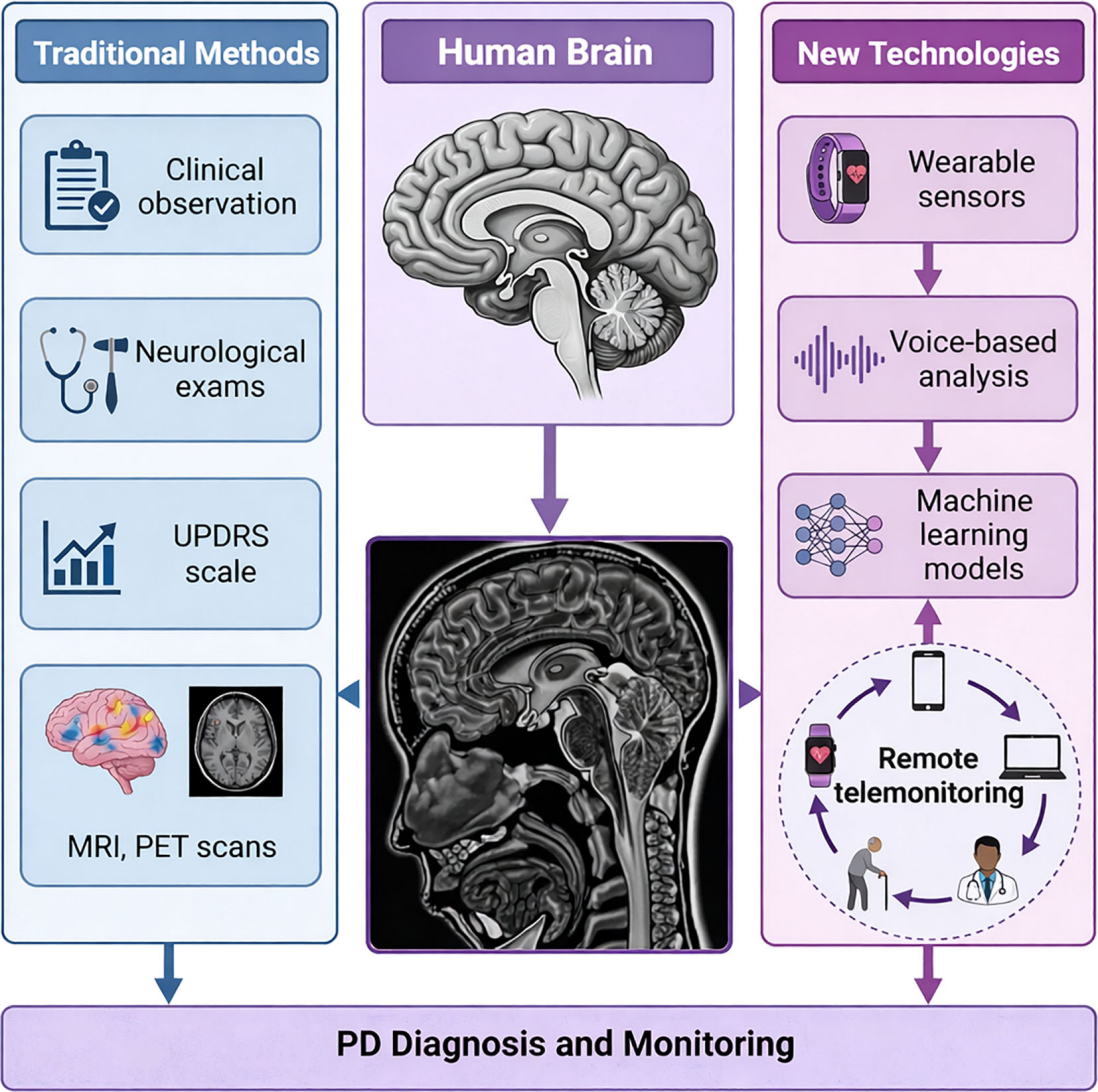
Conceptual framework integrating traditional clinical methods with AI-driven speech analysis for Parkinson’s disease diagnosis and continuous monitoring.

Using strict speaker-disjoint splits on the PC-GITA Spanish corpus (for training and validation) and an independent Mandarin clinical dataset (for testing), we demonstrate the model’s robust cross-lingual performance, noise tolerance, and interpretability. SHapley Additive exPlanations (SHAP) analysis confirms that the model’s predictions align with established biomarkers of dysarthria. This pioneering approach holds transformative potential for early detection, continuous remote monitoring, and personalized intervention, directly addressing the growing global PD crisis with innovative, non-invasive technology. To further test whether the learned representation transfers beyond the PC-GITA-to-Mandarin setting, we additionally evaluated the framework on Neurovoz, a public Castilian-Spanish Parkinsonian speech corpus comprising roughly 108–112 speakers with PD and healthy controls and a broad set of speech tasks, thereby providing a second external benchmark under different cohort characteristics and recording conditions. **Figure 1** provides the clinical and technological context for the study by contrasting conventional PD assessment pathways with the proposed AI-driven speech analysis workflow.

The workflow in **Figure 1** begins with traditional clinical methods on the left, including clinical observation, neurological examinations, UPDRS scale assessments, and neuroimaging techniques such as MRI and PET scans. These conventional approaches converge toward the central depiction of the human brain affected by Parkinson’s disease, which serves as the bridge between traditional and emerging technologies. The right panel demonstrates the AI-driven remote telemonitoring workflow, where voice recordings are captured via smartphones and wearable sensors, processed through machine learning models to extract multimodal speech biomarkers (MFCCs, jitter, shimmer, wavelet entropy, glottal parameters), and transmitted to healthcare providers for continuous monitoring. Such remote monitoring pipelines also depend on efficient sensing and data-routing strategies in distributed body-sensor environments, which have been studied as an enabling component of scalable digital health systems (Liu et al., 2020). This integrated framework highlights how the hybrid CNN-Mamba model complements traditional diagnostics, offering scalable, accessible, and cost-effective early detection and ongoing monitoring capabilities, particularly valuable in resource-limited settings.

## 2 Methods

**Figure 2:**
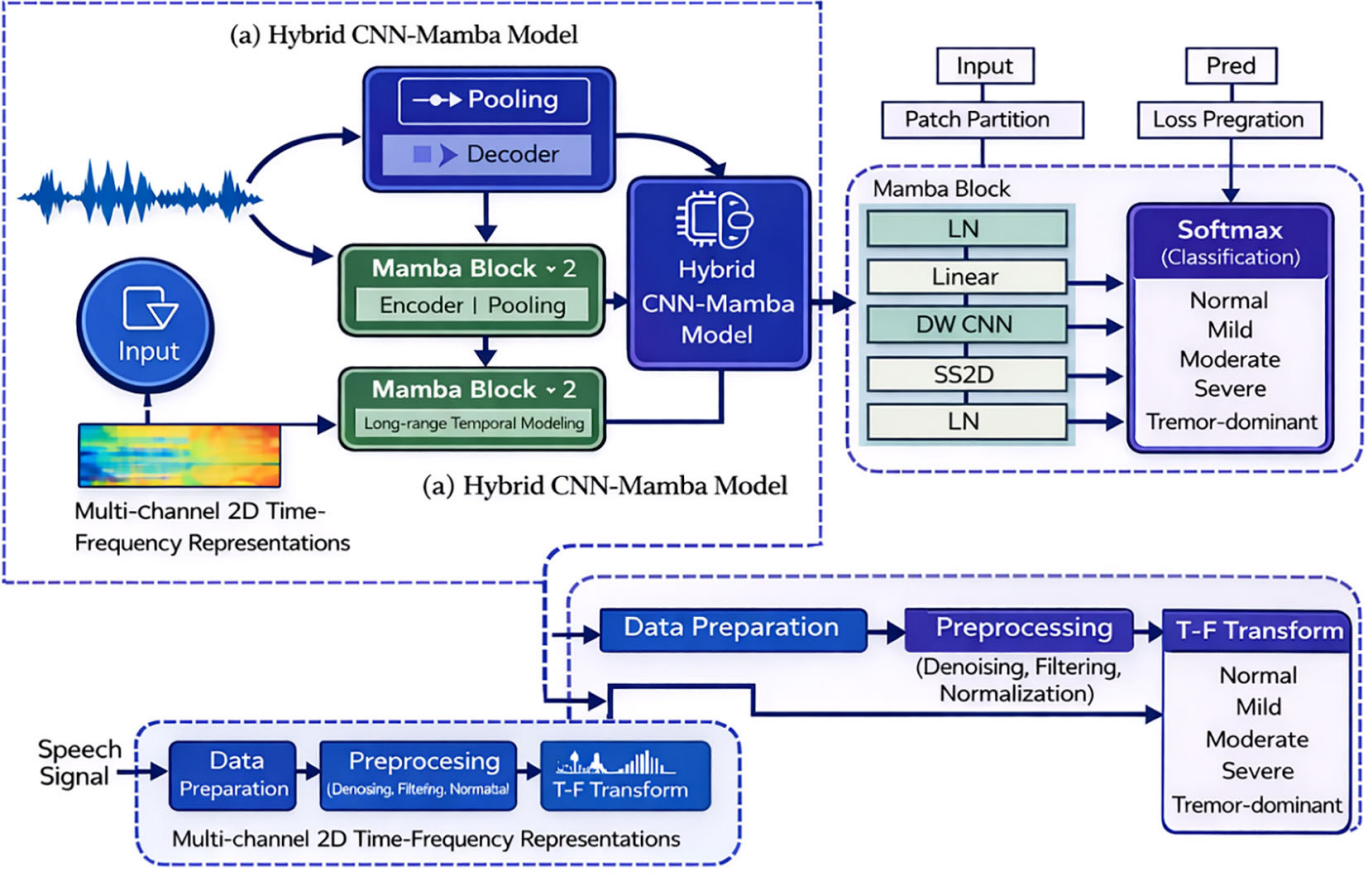
End-to-end hybrid CNN-Mamba architecture for multi-class PD severity classification.

This study presents an automatic multi-class classification framework for Parkinson’s disease (PD) speech impairment severity using a hybrid Convolutional Neural Network (CNN) and Mamba selective state space model (SSM) architecture. The CNN component captures local spatial and spectro-temporal patterns from multimodal acoustic representations, while Mamba blocks enable efficient, linear-time modeling of long-range temporal dependencies (e.g., progressive monotonicity, tremor modulation across utterances) without the quadratic complexity of attention mechanisms (Goel et al., 2022; Yadav and Tan, 2024). A final softmax layer yields probabilities over five clinically defined classes: Normal, Mild, Moderate, Severe, and the phenotype-informed Tremor-dominant subgroup. The Mish activation function is used throughout for enhanced training stability and non-linearity (Misra, 2019). **Figure 2** summarizes the complete processing pipeline, from preprocessed speech representations to final five-class prediction, and highlights how CNN-based local feature extraction is coupled with Mamba-based temporal modeling.

The illustration of **Figure 2** shows the complete end-to-end pipeline of the proposed hybrid CNN–Mamba framework, from raw speech signal acquisition to final severity classification. The workflow begins with the data preparation stage, where raw speech recordings undergo preprocessing steps, including normalization and noise handling, followed by a time–frequency (T–F) transformation to generate spectrogram-based representations. These spectrograms serve as the input to the hybrid model.

Within the Hybrid CNN–Mamba Model, convolutional neural network (CNN) components are employed to extract local spatial and spectro-temporal features from the input representations, enabling effective modeling of short-term acoustic characteristics associated with dysarthria, such as local energy variations and harmonic irregularities. The extracted feature maps are subsequently passed to stacked Mamba blocks, which perform efficient long-range temporal modeling. These blocks capture extended temporal dependencies and progression-related speech patterns relevant to Parkinson’s disease severity.

The Figure also details the internal structure of the Mamba block, consisting of layer normalization, linear projection, depthwise convolution, state space sequence modeling (SS2D), and residual connections, enabling stable and expressive temporal feature learning. The outputs from the CNN and Mamba components are fused within the hybrid architecture and forwarded to the classification head.

Finally, the fused features are processed through a softmax-based classification layer to predict one of five clinically defined Parkinson’s disease speech classes: Normal, Mild, Moderate, Severe, and the phenotype-informed Tremor-dominant subgroup. This architecture effectively integrates local spatial feature extraction with long-range temporal modeling, providing a robust and scalable solution for multi-class Parkinson’s disease severity assessment from speech signals. No wearable, kinematic, or autonomic sensor data were used in this study; all analyses were performed exclusively on recorded speech signals.

### 2.1 Mathematical formulation of the CNN-Mamba architecture

Let $\text{x}\in {\mathbb{R}}^{T\times D}$ denote a preprocessed speech feature sequence of length *T*(time steps) and dimension *D*(derived from 2D time frequency representations). The CNN backbone first extracts local spatial features are given in Equation (1):

(1) $${\text{h}}_{\text{CNN}}=\text{CNN}(\text{x};{\text{W}}_{c},{\text{b}}_{c},\phi_{\text{Mish}})$$

where ${\text{W}}_{c}$ and ${\text{b}}_{c}$ are the convolutional weights and biases, and $\phi_{\text{Mish}}$ is the Mish activation function defined in Equation (2) as:

(2) $$\phi_{\text{Mish}}(z)=z\cdot \text{tanh}(\text{ln}(1+e^{z}))$$

The CNN output ${\text{h}}_{\text{CNN}}\in {\mathbb{R}}^{T^{\prime }\times C}\;$ (downsampled sequence length $\;T^{\prime }$, number of channels *C*) is then fed into the Mamba blocks, which implement selective state space modeling (Yadav and Tan, 2024). The core discretized selective SSM dynamics are given by the following state and output equations are give in Equations (3) and in (4):

(3) $${\bar{\text{h}}}_{t}=\bar{\text{A}}\cdot {\bar{\text{h}}}_{t-1}+{\bar{\text{B}}}_{t}\cdot {\text{u}}_{t}$$

(4) $${\text{y}}_{t}={\text{C}}_{t}\cdot {\bar{\text{h}}}_{t}+\text{D}\cdot {\text{u}}_{t}$$

where ${\text{u}}_{t}$ is the input at time step *t*, and $\bar{\text{A}},{\bar{\text{B}}}_{t},{\text{C}}_{t}$ are input-dependent, discretized parameters computed selectively using the context-aware step size ${\text{Δ}}_{t}$ is given in Equation (5) as:

(5) $$\bar{\text{A}}=\text{exp}\left({\text{Δ}}_{t}⊙\text{A}\right),{\bar{B}}_{t}=\left({\text{Δ}}_{t}⊙{\text{B}}_{t}\right)\cdot \text{softplus}\left({\text{Δ}}_{t}\right)$$

With ⊙ denoting element-wise multiplication. The selective scan algorithm enables efficient parallel computation across the entire sequence (Goel et al., 2022; Yadav and Tan, 2024).

Multiple Mamba blocks are stacked, each incorporating residual connections and layer normalization. The final hidden representation from the Mamba stack, denoted $h_{\text{Mamba}}$, is globally pooled (typically global average pooling along the time dimension) and passed through a linear projection followed by a softmax layer in Equation (6) as:

(6) $$\hat{\text{p}}=\text{softmax}({\text{W}}_{o}\cdot \text{Pool}({\text{h}}_{\text{Mamba}})+{\text{b}}_{o})$$

where $\hat{p}$ represents the predicted probability distribution over the five speech impairment severity classes (Normal, Mild, Moderate, Severe, Tremor-dominant).

### 2.2 Data acquisition and preprocessing

#### 2.2.1 Data sources and recording details

Speech recordings were sourced from two independent datasets to ensure linguistic diversity, clinical variability, and robust evaluation of cross-lingual generalization. The PC-GITA dataset (Orozco-Arroyave et al., 2016), a publicly available Spanish-language corpus specifically designed for Parkinson’s disease speech research, includes recordings from 50 individuals with PD (31 male, 19 females; mean age 61.2 ± 9.3 years) and 50 age- and gender-matched healthy controls. The tasks in this dataset encompass sustained vowels (/a/held for at least 5 seconds), diadochokinetic sequences (/pa-ta-ka/,/pa-ka-ta/), isolated word repetitions, and sentence readings, capturing a wide spectrum of phonatory, articulatory, and prosodic deficits characteristic of hypokinetic dysarthria. The selection of sustained phonation, rapid syllable repetition, and connected/structured speech tasks is also consistent with published methodological guidance for acoustic assessment in dysarthrias of movement disorders (Rusz et al., 2021).

The internal clinical dataset was collected at Huaqiao University Affiliated Strait Hospital (ethical approval HQU-2021-047, conducted in accordance with local legislation and institutional requirements; written informed consent obtained from all participants or legal guardians). This dataset comprises Mandarin Chinese speech recordings from 126 PD patients (78 male, 48 females; age range 52–81 years, mean 67.3 ± 8.1 years; disease duration 1–15 years, mean 6.2 ± 3.8 years; UPDRS-III motor scores 18–68, mean 42.5 ± 12.3) and 63 age- and gender-matched healthy controls. Recordings in this dataset include sustained vowel/a/, reading of standardized Mandarin passages, and spontaneous speech elicited through open-ended prompts.

Data were acquired in sound-attenuated rooms (<35 dB ambient noise) using Shure SM58 microphones at a sampling rate of 44.1 kHz and then down-sampled to 16 kHz for cross-dataset consistency. Approximately 60% of PD participants were inpatients, and 40% were outpatients, ensuring representation across varying severity levels and real-world clinical conditions.

The PC-GITA data was used exclusively for training and 5-fold cross-validation, while the internal Mandarin dataset was reserved solely for independent final testing to rigorously evaluate generalization to unseen speakers, different languages, accents, and recording environments. **Table 1** summarizes the demographic and usage characteristics of the PC-GITA and Mandarin cohorts, establishing the differences in language, cohort size, and study role that motivate the cross-lingual evaluation design.

**Table 1:** Dataset characteristics.

Characteristic	PC-GITA dataset	Internal clinical dataset
Language	Spanish	Mandarin Chinese
PD Patients	50	126
Healthy Controls	50	63
Age (PD, mean ± SD)	61.2 ± 9.3 years	67.3 ± 8.1 years
Gender (PD, M: F)	31:19	78:48
Usage	Training/Validation	Testing only

#### 2.2.2 Preprocessing pipeline

To suppress noise, preserve dysarthria-relevant cues, and standardize inputs, the following preprocessing steps were applied sequentially to each raw audio segment. First, a 4-level discrete wavelet transform (DWT) was applied using the Daubechies-4 (db4) mother wavelet. This transformation helps suppress high-frequency noise while retaining low-frequency phonatory structure (Liu et al., 2022). Next, a 4th-order zero-phase Butterworth bandpass filter was used, with cutoff frequencies of 300–3400 Hz, to isolate the primary speech band and remove out-of-band artifacts (McFall et al., 2023). Additionally, zero-phase infinite impulse response (IIR) high-pass filtering with a cutoff frequency of 80 Hz was applied to correct for baseline wander. Finally, segment-wise Z-score normalization was performed to achieve zero mean and unit variance. The recording and analysis workflow was designed to align, as far as permitted by the retrospective public-corpus setting, with methodological recommendations for speech recording and acoustic analysis in movement-disorder dysarthrias (Rusz et al., 2021). The Z-score normalization formula is given Equation (7) as follows:

(7) $$x_{\text{norm}}(t)=\frac{x(t)-\mu }{\sigma }$$

where μ and σ represent the mean and standard deviation of the segment, respectively. This step ensures amplitude invariance across speakers, sessions, and recording devices, promoting stable gradient flow during training.

**Figure 3:**
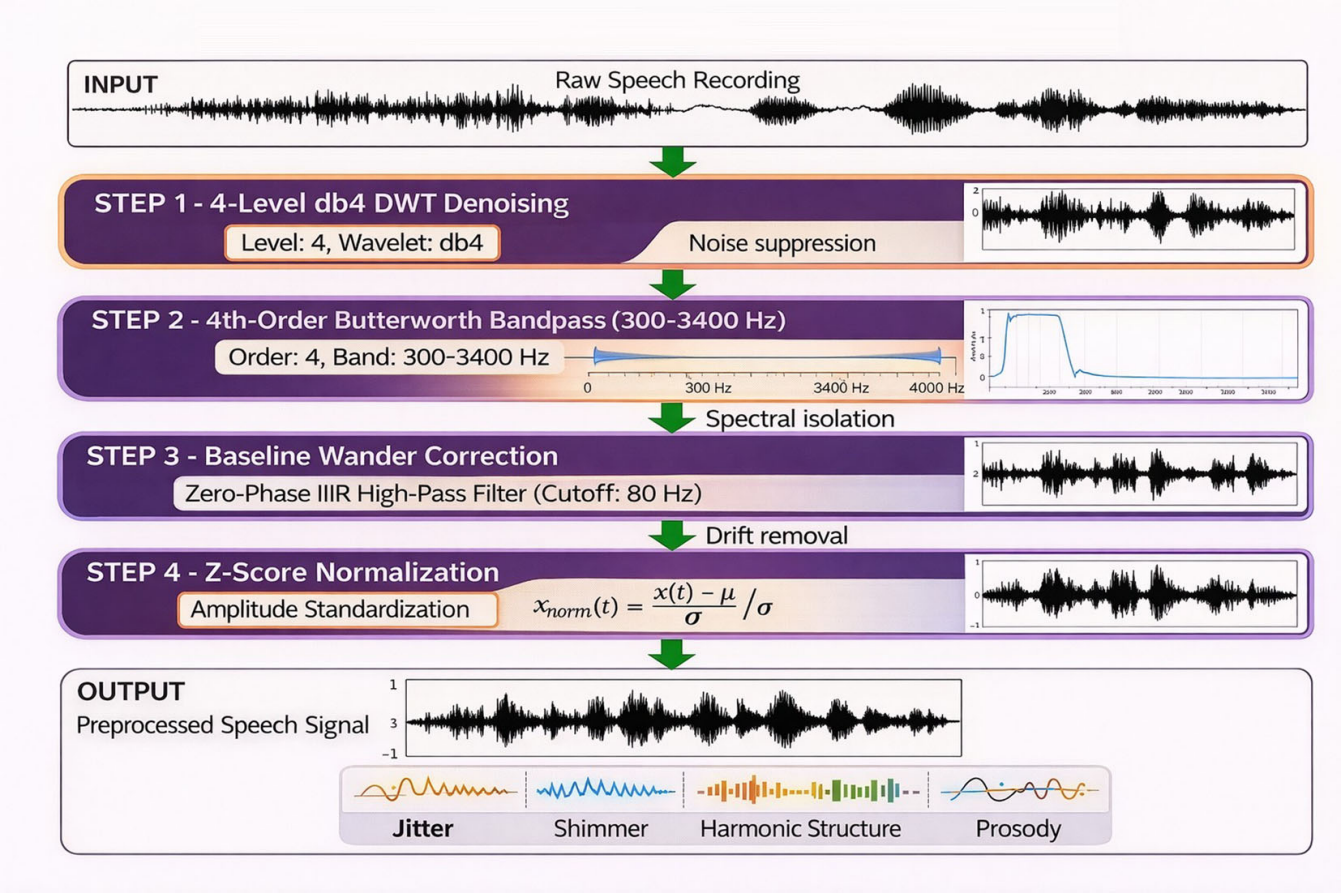
Signal preprocessing pipeline for PD speech analysis.

As illustrated in **Figure 3**, the preprocessing pipeline was designed to reduce noise while preserving dysarthria-relevant phonatory and spectral cues needed for downstream classification.

**Figure 3** shows the sequential preprocessing operations applied to raw speech data before feature extraction and model training. The flowchart shows four key steps: first, a 4-level db4 discrete wavelet transform is applied for denoising to suppress high-frequency noise while preserving low-frequency phonatory structures. Second, a 4th-order zero-phase Butterworth bandpass filter (300–3400 Hz) isolates the primary speech frequency band and removes out-of-band artifacts. Third, zero-phase IIR baseline wander correction eliminates low-frequency drift. Finally, segment-wise Z-score normalization standardizes amplitude across different speakers and recording conditions. Each preprocessing step is carefully designed to preserve dysarthria-specific acoustic cues such as jitter, shimmer, harmonic structure, and prosody, which are essential biomarkers for accurate PD severity classification in the subsequent CNN-Mamba modeling framework.

#### 2.2.3 Segmentation strategy

Each preprocessed audio file was divided into two complementary window lengths to capture both broad contextual/prosodic information and fine-grained phonatory perturbations. Specifically, 4-second windows were used to optimize for suprasegmental features such as intonation contours, speech rate, and long-term tremor modulation. In contrast, 0.75-second windows were used to capture short-term cycle-to-cycle perturbations (e.g., jitter, shimmer) that are prevalent in sustained vowels and rapid sequences. These windows were non-overlapping to avoid artificial correlations between segments. This dual-segmentation approach generated approximately 25,000 samples from the original 252 recordings.

To ensure data integrity, a strict speaker-disjoint splitting protocol was enforced. All segments belonging to any single speaker were assigned entirely to one partition (either training, validation, or test). This design eliminates data leakage from speaker-specific characteristics, such as unique vocal tract resonances or habitual speaking styles, and ensures true subject-independent performance, which is essential for the clinical translation of the model (Xiang et al., 2025).

#### 2.2.4 Additional external validation on the Neurovoz corpus

To strengthen external validation, we included the public Neurovoz corpus as an additional out-of-domain benchmark. Neurovoz is a Castilian-Spanish Parkinsonian speech dataset reported as containing approximately 108–112 native speakers, including roughly balanced groups of individuals with Parkinson’s disease and healthy controls, with recordings collected across multiple structured tasks such as sustained vowels, diadochokinetic tests, repeated utterances, and spontaneous/monologic speech. Because Neurovoz does not natively provide the five-class severity framework used in the present study, it was employed as an auxiliary external binary validation corpus. For this analysis, the Normal class was retained as the control class and all Parkinsonian subclasses learned in the main framework were collapsed into a single PD category for external transfer testing. No Neurovoz speakers were used in model training.

### 2.3 Five-class labeling, severity mapping, and balancing

The classification task was defined over five clinically defined classes to enable assessment of both speech-impairment severity and phenotype-aware subgroup differentiation. These classes are as follows: Normal (N), which includes recordings from healthy controls with no clinical diagnosis of Parkinson’s disease or perceptible dysarthria; Mild (M), corresponding to mild perceptual dysarthria and aligning with a score of 1 on the UPDRS-III speech item 3.1, indicating slight reduction in volume, mild monotone quality, or subtle articulatory imprecision; Moderate (Mo), which represents moderate perceptual dysarthria, with a score of approximately 2 on the UPDRS speech assessment, characterized by noticeable hypophonia, monotone prosody, and reduced intelligibility; Severe (S), indicating severe perceptual dysarthria with a UPDRS speech score between 3 and 4, marked by significantly reduced intelligibility, near-anarthric features, or significant prosodic flattening; and Tremor-dominant (T), a phenotype-informed PD subgroup rather than an ordinal speech-severity stage. This subgroup was defined by a resting/postural tremor score (sum of UPDRS items 20 and 21) at least 1.5 times higher than the rigidity score (UPDRS item 22). Because the PD speech literature provides stronger evidence for hypokinetic dysarthria biomarkers than for a uniquely separable Tremor-dominant acoustic phenotype, this subgroup is interpreted here as exploratory phenotype-aware classification rather than as a point on the same ordinal severity continuum as Mild, Moderate, and Severe.

The original PC-GITA corpus provides binary diagnostic labels (Parkinson’s disease versus healthy control) rather than native multi-class speech-severity annotations. To construct the present five-class framework, PD speakers were re-annotated at the speaker level into Mild, Moderate, Severe, and Tremor-dominant subclasses, while healthy controls retained the Normal label. All recordings and derived segments from the same speaker inherited the same consensus label. For the PC-GITA cohort, Mild, Moderate, and Severe labels were assigned independently by two certified speech-language pathologists with experience in movement-disorder dysarthria through blinded auditory-perceptual evaluation of the available speech material for each speaker, including sustained phonation, syllable repetition, word/sentence production, and connected speech. Because item-specific UPDRS-III speech scores were not available from the public PC-GITA release, the raters did not perform direct matching to ground-truth UPDRS values. Instead, they used UPDRS-informed perceptual anchors: Mild denoted subtle but consistent speech abnormality with preserved intelligibility; Moderate denoted clearly abnormal speech with reduced articulatory/prosodic control and reduced communicative clarity; Severe denoted marked dysarthric impairment with substantial intelligibility loss, severely flattened prosody, or near-anarthric characteristics. Initial inter-rater agreement was assessed before consensus using Cohen’s kappa and yielded κ = 0.78. Disagreements were resolved through consensus discussion, with neurologist input when required. The Tremor-dominant category was treated separately as a phenotype-informed subgroup; in the Mandarin clinical cohort, Tremor-dominant status was identified by neurologists using available UPDRS-III motor data, defined as a tremor score (items 20 and 21) at least 1.5 times higher than the rigidity score (item 22).

We emphasize that Tremor-dominant was not treated as a strict ordinal speech-severity level equivalent to Mild, Moderate, or Severe. Rather, it was included as a phenotype-informed subgroup to explore whether motor subtype information may be associated with distinguishable acoustic behavior in structured speech. The most plausible candidate cues are rhythmic amplitude or frequency modulation, low-frequency instability, phonatory periodicity changes, and tremor-related modulation superimposed on otherwise dysarthric speech. However, because direct evidence for a uniquely separable Tremor-dominant speech phenotype remains limited, the present results should be interpreted as exploratory phenotype-aware classification rather than definitive proof of a standalone tremor-specific speech class.

Prior to formal annotation, the two speech-language pathologists jointly reviewed a calibration subset of representative PD recordings to harmonize the interpretation of severity boundaries, especially the Mild versus Moderate boundary, which is perceptually ambiguous in hypokinetic dysarthria. During independent labeling, the raters considered five primary auditory-perceptual dimensions: (i) vocal quality abnormality, including roughness or breathiness; (ii) articulatory imprecision; (iii) reduction in prosodic variation or monopitch; (iv) speech rate abnormality or rhythm instability; and (v) overall intelligibility. Mild cases showed subtle but repeatable abnormality with preserved communicative function; Moderate cases showed clearly abnormal speech with reduced clarity and prosodic control; Severe cases showed pronounced impairment with substantial intelligibility loss or unstable, effortful, or near-anarthric output.

The original speaker-level class distributions used in this study are reported explicitly to distinguish true cohort composition from post-segmentation balancing. In the PC-GITA cohort (100 speakers total), the class distribution was: Normal = 50, Mild = 18, Moderate = 15, Severe = 10, and Tremor-dominant = 7 speakers. In the Mandarin cohort (189 speakers total), the class distribution was: Normal = 63, Mild = 46, Moderate = 38, Severe = 26, and Tremor-dominant = 16 speakers. Thus, the Tremor-dominant subgroup comprised 7 speakers in PC-GITA and 16 speakers in the Mandarin cohort. To address the resulting segment-level class imbalance during training, the training split only was balanced to approximately 5,000 segments per class through targeted oversampling of minority classes. To preserve the strict no-leakage protocol, oversampling was performed by duplicating entire speaker blocks within the training partition only, ensuring that no cross-contamination occurred between the training, validation, or test partitions. We note, however, that this strategy may increase repeated exposure to speaker-specific acoustic profiles in minority classes and therefore may favor partial memorization of stable speaker characteristics rather than fully generalizable dysarthria-related patterns. **Table 2** reports the original speaker-level class distribution before oversampling, making clear that the minority subclasses, especially Tremor-dominant, were represented by substantially fewer speakers than the Normal and Mild groups.

**Table 2:** Original speaker-level class distribution in the PC-GITA and Mandarin cohorts before segment-level oversampling.

Class	PC-GITA (speakers)	Mandarin (speakers)
Normal	50	63
Mild	18	46
Moderate	15	38
Severe	10	26
Tremor-dominant	7	16
Total	100	189

During training, weighted categorical cross-entropy loss was employed to further compensate for residual imbalance, which is given in Equation (8) as follows:

(8) $$L=-\sum_{i=1}^{5}w_{i}\;y_{i}\text{log}({\hat{y}}_{i})$$

where $y_{i}$ is the one-hot encoded ground-truth label for the class *i*, ${\hat{y}}_{i}$ is the predicted probability, and the class weight $w_{i}$ is inversely proportional to class frequency in the training set as given in Equation (9) as follows:

(9) $$w_{i}=\frac{1}{f_{i}}$$

with $f_{i}\;$ denoting the relative frequency of a class *i*. This formulation ensures equitable gradient contribution from all severity levels during optimization (Rusz et al., 2021). In future work, this speaker-block duplication strategy will be compared against augmentation-based balancing approaches such as SpecAugment, time warping, and synthetic feature-space augmentation to determine whether similar minority-class recall can be achieved with less risk of overfitting to speaker identity. To clarify how the ordinal labels were operationalized, **Table 3** summarizes the auditory-perceptual criteria used to distinguish Normal, Mild, Moderate, and Severe speech impairment, while also separating the phenotype-informed Tremor-dominant subgroup from the ordinal severity continuum.

**Table 3:** Auditory-perceptual criteria for the ordinal severity classes and clinical interpretation of the Tremor-dominant subgroup in the PC-GITA framework.

Class	Auditory-perceptual criteria	Functional interpretation
Normal	No perceptible dysarthric abnormality	Healthy control speech
Mild	Subtle roughness/breathiness, slight monotone quality, mild articulatory imprecision, intelligibility preserved	Early or limited dysarthric involvement
Moderate	Clearly abnormal articulation and prosody, noticeable hypophonia, reduced clarity, intermittent intelligibility reduction	Clinically evident dysarthria
Severe	Marked phonatory/articulatory instability, severe prosodic flattening, substantial intelligibility loss, near-anarthric features	Advanced dysarthric impairment
Tremor-dominant	Phenotype-informed subgroup defined by neurologist-rated tremor predominance rather than ordinal speech severity alone	Motor-phenotype-associated PD subgroup

**Table 4** outlines the re-annotation workflow for PC-GITA, including rater expertise, independent blinded scoring, consensus resolution, and the observed inter-rater agreement.

**Table 4:** Annotation workflow and inter-rater agreement for PC-GITA re-labeling.

Item	Value
Original PC-GITA labels	Binary (PD vs. healthy control)
Re-annotation unit	Speaker level
Number of raters	2
Rater expertise	Certified speech-language pathologists
Blinded independent rating	Yes
Calibration subset reviewed before formal labeling	Yes
Initial Cohen’s kappa	0.78
Consensus resolution performed	Yes
Neurologist input for difficult cases	Yes

### 2.4 Feature extraction and 2D input formation

Following preprocessing, multimodal acoustic features were extracted from each segmented speech window to capture the diverse manifestations of hypokinetic dysarthria. These features include phonatory instability, such as jitter and shimmer, spectral envelope distortions through Mel-Frequency Cepstral Coefficients (MFCCs), irregularity through wavelet entropy, glottal source dysfunction, and noise intrusion measured by Harmonic-to-Noise Ratio (HNR). Feature extraction was performed using well-established open-source tools: Praat (v6.4) for time-domain perturbation measures and glottal parameters, openSMILE (v3.0) for spectral features and entropy, and COVAREP (v1.4.2) for glottal flow estimation. For the Tremor-dominant subgroup, the most plausible candidate cues were presumed to include low-frequency rhythmic modulation, amplitude instability, and tremor-related perturbation patterns superimposed on standard dysarthria markers; however, these were treated as exploratory rather than established phenotype-specific biomarkers.

The extracted feature set comprised several elements. The MFCCs included 13 static coefficients together with their first- and second-order temporal derivatives, computed using 25 ms Hamming windows with a 10 ms hop size. These coefficients capture formant structure, spectral tilt, and dynamic envelope changes associated with imprecise articulation and breathiness. In addition, perturbation features were extracted for each segmented analysis window, i.e., for the same speech segment that subsequently contributed to the multi-channel 2D input representation. Using Praat (v6.4), we computed local jitter, relative average perturbation (RAP), and the 5-point period perturbation quotient (PPQ5) to quantify cycle-to-cycle instability of the fundamental period, together with local shimmer (dB), APQ3, and APQ11 to quantify amplitude perturbation linked to irregular vocal-fold vibration. For sustained-vowel and other highly periodic windows, these measures were computed in the conventional manner. For reading and sentence-level material, perturbation features were derived from the voiced portions within each segmented window, rather than from unvoiced intervals, so that the reported values retained their interpretation as phonatory-instability descriptors. Wavelet entropy was computed as Shannon entropy over a 4-level db4 wavelet decomposition to characterize signal irregularity and non-periodicity. Glottal source parameters, including the open quotient (OQ), glottal flow quotient (GFQ), and the first harmonic-to-second harmonic ratio (H1-H2), were extracted to reflect laryngeal dysfunction and breathy voice quality. Finally, the Harmonic-to-Noise Ratio (HNR) was used to quantify the balance between periodic and aperiodic energy in the speech signal.

To prepare the data for CNN input, the perturbation and other scalar descriptors extracted from each segmented window, including jitter and shimmer, were frame-aligned or interpolated to the same temporal grid as the MFCC sequence (25 ms frames, 10 ms hop). These aligned descriptors were then stacked as additional channels alongside the MFCC-based representation, yielding a multimodal tensor in which all channels corresponded to the same underlying speech segment. The resulting time × feature-dimension × channel representation was subsequently resized to a fixed 128 × 128 × C format (typically C = 20–25) using bilinear interpolation, thereby preserving the relative spatial organization of the multimodal features while enabling standardized CNN input.

This fusion strategy allowed the CNN backbone to learn joint spatio-temporal representations of local dysarthria patterns, such as harmonic disruptions in specific frames, without requiring manual feature selection or dimensionality reduction.

### 2.5 Convolutional neural network backbone

Convolutional neural networks (CNNs) are particularly effective at capturing local spatial and spectro-temporal patterns in structured 2D inputs, such as time-frequency representations, making them ideally suited for processing the multi-channel speech feature maps generated in Section 2.4. In the hybrid CNN-Mamba architecture, the CNN backbone functions as the initial feature extractor, hierarchically learning representations of vocal fold vibration irregularities, formant transitions, harmonic disruptions, and perturbation clusters that characterize PD dysarthria.

The CNN backbone consists of four sequential convolutional blocks, each with progressively increasing channel depth. The first block has 32 filters, with 3×3 kernels, a stride of 1, and padding of 1. The second block uses 64 filters with 3×3 kernels. The third block expands to 128 filters with 3×3 kernels, and the fourth block has 256 filters with 3×3 kernels. Each convolutional layer is followed by the Mish activation function, which applies a non-linear transformation to preserve subtle negative signal components, such as spectral residuals indicative of noise intrusion. The Mish function is defined given in Equation (10) as:

(10) $$\phi_{\text{Mish}}(z)=z\cdot \text{tanh}(\text{ln}(1+e^{z}))$$

Mish was chosen over ReLU due to its smoother gradient propagation and superior performance on noisy, imbalanced biomedical time-series data (Misra, 2019; Pah et al., 2022). Following the activation, batch normalization is applied to stabilize activations, reduce internal covariate shift, and accelerate convergence. Batch normalization is computed in Equation (11) as:

(11) $${\hat{x}}_{b,i}=\frac{x_{b,i}-\mu_{B}}{\sqrt{\sigma_{B}^{2}+\epsilon }}\cdot \gamma +\beta \;$$

where $\mu_{B}\;$ and $\sigma_{B}^{\;2}$ are the batch mean and variance, γ and β are learnable scale and shift parameters, and $\epsilon =10^{-5}$is a small constant for numerical stability. To introduce spatial downsampling and ensure translation invariance critical for speaker-independent generalization a 2×2 max-pooling layer with a stride of 2 is applied after each convolutional block. Additionally, dropout with a probability of 0.1 is used after the pooling layers to prevent overfitting to dataset-specific artifacts.

**Figure 4:**
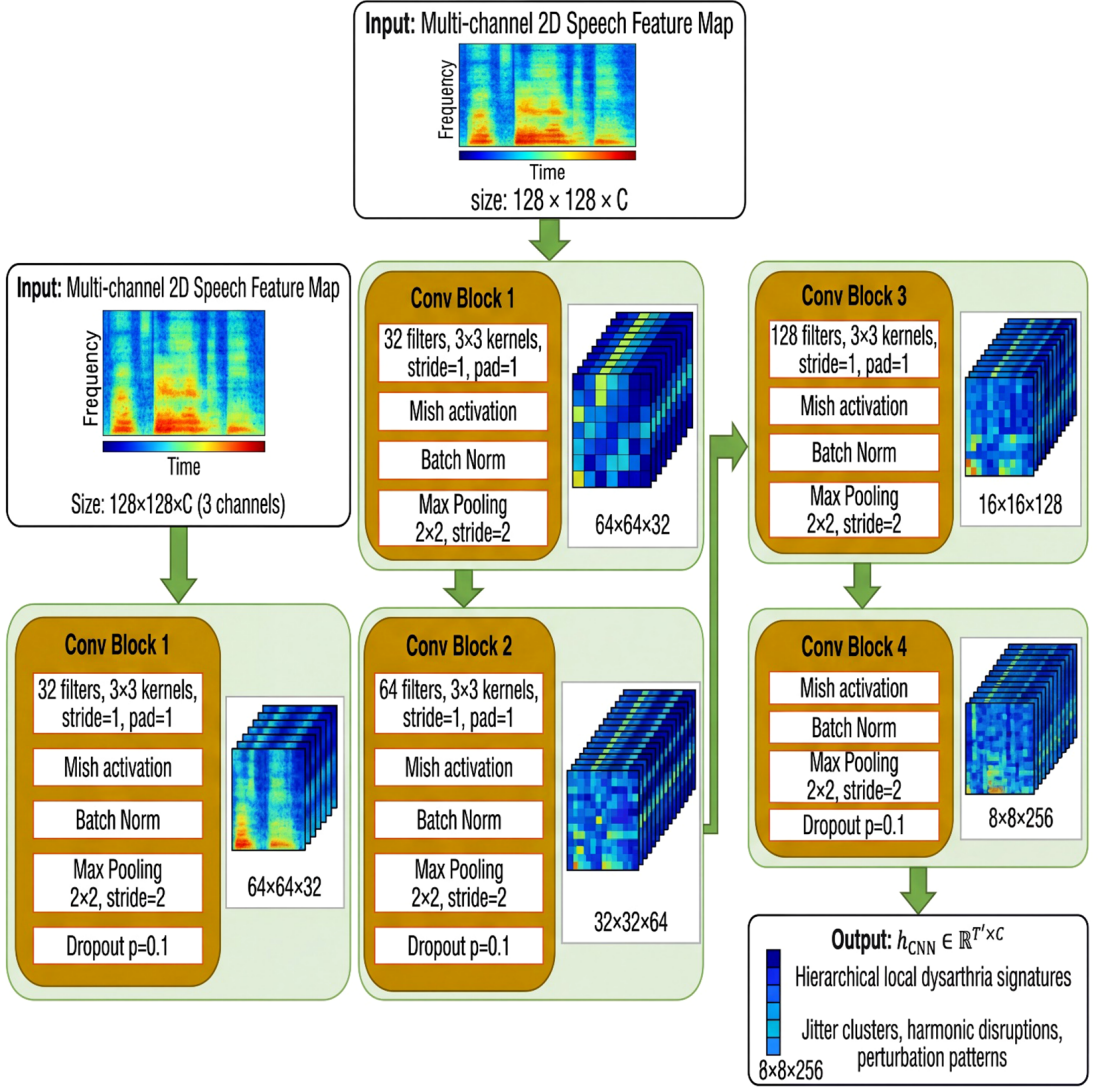
CNN-Mamba architecture. The fused acoustic representation is provided to the CNN as a 128 × 128 × C tensor, where C corresponds to the number of stacked feature channels derived from the multimodal speech descriptors.

The CNN progressively reduces spatial dimensions (e.g., from 128×128 to 8×8) while expanding channel depth (from 3 to 256), producing a downsampled feature sequence $h_{\text{CNN}}\in {\mathbb{R}}^{T^{\prime }\times C}$, as shown in, that encodes hierarchical local dysarthria signatures. This output is then passed directly to the Mamba blocks (described in Section 2.6) for long-range temporal integration. **Figure 4** expands on the feature-extraction stage by showing the internal organization of the CNN component and the progressive transformation of the multimodal input representation.

The illustration in **Figure 4** presents the detailed structure of the CNN backbone within the hybrid CNN-Mamba framework. The architecture processes multi-channel 2D speech feature maps (size 128×128×C channels) through four sequential convolutional blocks with progressively increasing filter depths (32→64→128→256 filters). Each block employs 3×3 kernels with stride=1 and pad=1, followed by Mish activation for enhanced non-linearity, batch normalization for training stability, and 2×2 max pooling with stride=2 for spatial downsampling. Dropout regularization (p=0.1) is applied after pooling layers to prevent overfitting. The CNN progressively reduces spatial dimensions from 128×128 to 8×8 while expanding channel depth to 256, extracting hierarchical local spectro-temporal dysarthria signatures including jitter clusters, harmonic disruptions, and formant irregularities. The final output is a downsampled feature sequence that encodes rich local patterns, ready for subsequent long-range temporal modeling by the Mamba blocks.

### 2.6 Mamba blocks and selective state space modeling

While the CNN backbone excels at extracting spatially local and morphologically rich features from 2D time-frequency representations, speech signals in Parkinson’s disease are inherently sequential and exhibit long-range dependencies. These dependencies include progressive monotonicity across utterances, gradual amplitude decay in hypophonia, and rhythmic tremor modulation spanning multiple seconds. Traditional recurrent architectures, such as LSTM and GRU, suffer from vanishing and exploding gradients over long sequences. Transformer-based models, on the other hand, incur quadratic computational and memory costs with sequence length, which limits their scalability for real-world, variable-length speech recordings (Erol et al., 2024; Wang et al., 2026).

To address these limitations, the proposed framework replaces recurrent or attention-based temporal modeling with Mamba, a selective state space model (SSM) that achieves linear-time complexity while maintaining or surpassing the expressive power of Transformers on long sequences (Goel et al., 2022; Yadav and Tan, 2024). The key innovation of Mamba is input-dependent selective parameterization, which allows the model to dynamically choose which parts of the input history to attend to. This enables efficient capture of extended contextual dependencies without the need for full quadratic attention.

Each Mamba block consists of several components. First, a linear projection is applied to expand the input dimensionality with an expansion factor of 2. This is followed by a depthwise convolution with a kernel size of 4 for local mixing. SiLU (Sigmoid Linear Unit) gating is then applied to modulate the selective path, and the core selective SSM layer uses input-dependent discretization of the continuous-time SSM parameters. The block also includes a residual connection and layer normalization to stabilize learning.

The selective SSM is defined in a discretized form as follows. Given input $u_{t}\in {\mathbb{R}}^{\text{dat}}$ at time step *t*, the model computes the context-aware step size ${\text{Δ}}_{t}\in {\mathbb{R}}^{N}$, where *N* is the state dimension (in this case, N=16), and discretizes the continuous SSM parameters, which are as given in Equation (12) as follows:

(12) $${\bar{\text{A}}}_{t}=\text{exp}\left({\text{Δ}}_{t}⊙A\right),{\bar{B}}_{t}=\left({\text{Δ}}_{t}⊙B_{t}\right)\cdot \text{softplus}\left({\text{Δ}}_{t}\right)$$

The hidden state evolves according to the discretized state Equation (13) as follows:

(13) $${\bar{h}}_{t}={\bar{A}}_{t}\cdot {\bar{h}}_{t-1}+{\bar{B}}_{t}\cdot u_{t}$$

and produces output through Equation (14) as:

(14) $$y_{t}=C_{t}\cdot {\bar{h}}_{t}+D\cdot u_{t}$$

The selective scan algorithm computes this recurrence in parallel across the entire sequence using associative scan operations, achieving O(L) time complexity (where *L* is the sequence length) compared to $O(L^{2})$ for attention or O(L) for sequential recurrence in RNNs (Yadav and Tan, 2024). Four Mamba blocks are stacked, each with residual connections, forming the final architecture as given in Equation (15):

(15) $$h_{\text{out}}=\text{LayerNorm}(h_{\text{in}}+\text{Mamba}(\text{LayerNorm}(h_{\text{in}})))$$

This design enables the model to efficiently integrate long-range temporal context, such as utterance-level monotonicity or tremor periodicity, while remaining hardware-efficient and suitable for edge deployment (Goel et al., 2022; Zhou et al., 2025).

### 2.7 Experimental settings

The hybrid CNN-Mamba model was implemented in PyTorch 2.3.0. All development, training, validation, ablation, and final evaluation reported in this manuscript were conducted on a high-performance workstation with the following configuration: Intel Core i9-13900K CPU (24 cores/32 threads), 64 GB DDR5 RAM, and an NVIDIA RTX 4090 GPU (24 GB GDDR6X VRAM) running Ubuntu 22.04 LTS with CUDA 12.1 and cuDNN 8.9. Python 3.10.12 was used for all development. Mixed precision training (torch.cuda.amp) was enabled to accelerate computation and reduce memory usage while maintaining numerical stability. The final model comprises approximately 1.1 million trainable parameters and requires around 8 million FLOPs per inference pass. On the RTX 4090 GPU, the average inference time was 9 ms per sample (batch size 1), confirming its suitability for real-time clinical applications and potential deployment on edge devices such as smartphones. All experiments were repeated three times with different random seeds (42, 123, and 456) to report mean ± standard deviation.

### 2.8 Training protocol, hyperparameters, and optimization

The hybrid CNN-Mamba model was trained end-to-end using supervised learning on the speaker-disjoint training partitions of the PC-GITA dataset. All experiments were conducted in PyTorch 2.3.0 on the hardware described in Section 2.7, namely an NVIDIA RTX 4090 GPU (24 GB GDDR6X VRAM) with CUDA 12.1.

For optimization, the AdamW optimizer was chosen, with a weight decay of 0.01 to regularize large weights and improve generalization (Zhao et al., 2022). The initial learning rate was set to 0.001 and decayed using cosine annealing with warm-up, defined by the following Equation (16):

(16) $$\eta_{t}=\eta_{\text{min}}+\frac{1}{2}(\eta_{\text{max}}-\eta_{\text{min}})(1+\text{cos}(\frac{t}{T_{\text{max}}}\pi ))$$

where $\eta_{\text{max}}=0.001$, $\eta_{\text{min}}=10^{-6}$, and $T_{\text{max}}$ is the total number of training steps, with warm-up applied over the first 10% of the steps. The loss function employed was weighted categorical cross-entropy, which addressed class imbalance. This loss function is defined in Equation (17) as:

(17) $$L(y,\hat{y})=-\sum_{i=1}^{5}w_{i}\;y_{i}\text{log}({\hat{y}}_{i})$$

where the inverse-frequency class weights $w_{i}=1/f_{i}$ ensure a balanced contribution from all severity levels, with particular emphasis on improving recall for the Severe and Tremor-dominant classes. Key training hyperparameters were optimized via grid search over 5-fold cross-validation on the PC-GITA dataset.

The main optimization settings used across experiments are summarized in **Table 5** to facilitate reproducibility and clarify the training regime applied during model development.

**Table 5:** Key training hyperparameters.

Parameter	Value	Rationale
Batch size	64	Balances memory usage and gradient stability
Initial learning rate	0.001	Standard for AdamW on speech tasks
Weight decay	0.01	Regularization to prevent overfitting
Dropout (CNN blocks)	0.1	After pooling layers
Dropout (FC layer)	0.3	Before final projection
Epochs (maximum)	200	With early stopping
Early stopping patience	15 epochs	Monitored on validation MCC
Gradient clipping	max norm = 1.0	Prevents exploding gradients in long sequences
Mixed precision (AMP)	Enabled	Faster training, lower memory footprint
Random seed	42	For reproducibility

The models were trained with batch shuffling within speaker-disjoint folds, and early stopping was applied when the validation Matthews Correlation Coefficient (MCC) did not improve for 15 consecutive epochs. Key hyperparameters are highlighted in **Table 5**. The best model per fold was selected based on the highest validation MCC and evaluated on the held-out Mandarin test set. To assess the contribution of individual components, systematic ablation studies were performed under identical conditions. These included testing the model with the CNN backbone alone, where Mamba blocks were removed; using Mamba-only, where the CNN was replaced by direct input to the Mamba model; replacing Mamba with a bidirectional LSTM (CNN-LSTM); substituting Mamba with a 4-layer Transformer encoder (CNN-Transformer); using ReLU as the activation function instead of Mish; and replacing the selective state space model (SSM) with the standard SSM. These experiments were designed to quantify the advantages of the selective SSM over recurrent and attention-based alternatives, as well as the performance difference between Mish and ReLU (Misra, 2019; Ayoub et al., 2023). For the per-class cross-corpus analysis, class-wise recall values were also retained separately for each random seed (42, 123, and 456) to assess whether apparent cross-dataset differences reflected stable trends or run-to-run variability. In future analyses, the Tremor-dominant subgroup will be more appropriately evaluated as a dedicated binary phenotype-identification task (Tremor-dominant versus non-Tremor-dominant PD), rather than being interpreted as part of a purely ordinal severity hierarchy. The retained fold-wise and seed-wise predictions were also used for paired statistical comparison between CNN-Mamba and baseline models at the speaker level.

### 2.9 Evaluation metrics and performance assessment

Model performance was assessed using a comprehensive set of metrics tailored for multi-class, imbalanced biomedical classification. These metrics included accuracy, which reflects the overall correct predictions; macro-averaged F1-score, which is the harmonic mean of precision and recall, equally weighted across classes to avoid bias toward the majority class; sensitivity (recall) and specificity, both computed per class and macro-averaged; and the Matthews Correlation Coefficient (MCC), a balanced metric that is robust to class imbalance. The MCC is defined in Equation (18) as:

(18) $$\text{MCC}=\frac{TP\cdot TN-FP\cdot FN}{\sqrt{\left(TP+FP)(TP+FN)(TN+FP)(TN+FN\right)}}$$

where TP, TN, FP, and FN represent the true positives, true negatives, false positives, and false negatives, respectively. Additionally, ROC-AUC (macro-averaged) was used to measure the area under one-vs-rest ROC curves. All metrics were computed on the independent Mandarin test set after hyperparameter tuning using 5-fold cross-validation on the PC-GITA dataset. To further interpret the model’s predictions, confusion matrices, per-class ROC curves, and SHAP value analysis (described in Section 3.5) were employed to identify the contributions of individual features. Evaluation was performed using accuracy, macro-F1, recall, specificity, MCC, ROC-AUC, and speaker-level confusion matrices, which were used to visualize the distribution of class-specific errors across the five clinically defined classes.

To assess whether performance differences between CNN-Mamba and the baseline models were statistically meaningful, additional paired significance testing was performed using speaker-level performance rather than segment-level outputs, thereby avoiding inflation from correlated within-speaker segments. For each fold and seed, speaker-level predictions from the proposed model and each baseline were compared using a paired permutation test on macro-F1 and accuracy. In addition, 95% bootstrap confidence intervals were estimated for the primary speaker-level metrics. These analyses were used to determine whether the observed improvements over CNN-only and CNN-BiLSTM reflected consistent model advantages beyond run-to-run variability.

In addition to the primary five-class evaluation on PC-GITA and the Mandarin cohort, the final model was also tested on the public Neurovoz corpus as an auxiliary external PD-versus-control benchmark. This additional analysis was designed to test whether the learned representation transfers to a second public corpus with different participants, tasks, and recording conditions, even though the full five-class severity framework could not be reproduced on that dataset because equivalent class annotations are not natively available.

Because each speaker contributed multiple correlated speech segments, performance was additionally evaluated at the speaker level to reflect the clinically relevant task of classifying an unseen subject rather than individual windows. For each speaker, all segment-level predictions were aggregated into a single subject-level prediction. The primary aggregation rule was majority voting over predicted segment labels. As a supplementary robustness analysis, a second subject-level prediction was obtained by averaging the posterior class probabilities across all segments from the same speaker and selecting the class with the highest mean probability. Speaker-level accuracy, macro-F1, macro-recall, specificity, MCC, and confusion matrices were computed for both the PC-GITA validation folds and the independent Mandarin test cohort. Segment-level metrics were retained as secondary analyses for methodological comparison.

## 3 Results

This section presents the quantitative performance of the proposed CNN-Mamba framework, including classification accuracy, cross-lingual generalization, ablation studies, and interpretability analysis via SHAP. All final metrics were computed on the independent Mandarin clinical test set. Validation results from 5-fold cross-validation on PC-GITA are included for comparison and internal consistency. All values are reported as mean ± standard deviation over three independent runs with different random seeds.

### 3.1 Classification performance

The CNN-Mamba model was evaluated at both the segment level and the speaker level. Because the segmentation strategy generates multiple correlated windows from the same subject, speaker-level evaluation was treated as the primary clinically meaningful endpoint, while segment-level results were retained as secondary analyses for methodological comparison. As shown in **Table 6**, segment-level performance reached 97.8% on PC-GITA and 95.4% on the Mandarin cohort. After aggregating predictions within each speaker, the model achieved 94.0% accuracy on PC-GITA and 91.2% on Mandarin using majority voting. Mean-probability aggregation provided slightly higher speaker-level performance, reaching 94.8% and 91.9%, respectively. These findings indicate that the reported gains are not solely attributable to repeated within-speaker segment predictions. The original speaker-level class counts for both datasets are reported in **Table 2** to distinguish cohort composition from the balanced segment-level training distribution. **Table 6** compares segment-level and speaker-level performance, showing that the proposed model maintains strong accuracy after clinically relevant speaker-level aggregation.

**Table 6:** Comparison of segment-level and speaker-level performance using majority-vote and mean-probability aggregation.

Dataset	Evaluation level	Aggregation	Accuracy (%)	Macro-F1	Macro-recall	Specificity	MCC
PC-GITA	Segment-level	—	97.8	0.97	0.97	0.99	0.95
PC-GITA	Speaker-level	Majority vote	94.0	0.93	0.93	0.98	0.89
PC-GITA	Speaker-level	Mean probability	94.8	0.94	0.94	0.98	0.90
Mandarin	Segment-level	—	95.4	0.95	0.95	0.98	0.91
Mandarin	Speaker-level	Majority vote	91.2	0.90	0.90	0.96	0.84
Mandarin	Speaker-level	Mean probability	91.9	0.91	0.91	0.97	0.88

Speaker-level per-class recall remained strongest for the Normal and Severe categories, while most residual errors occurred between Mild and Moderate and between Moderate and Tremor-dominant. This pattern is clinically plausible because adjacent or partially overlapping symptom profiles are more difficult to separate than the extremes of the severity continuum.

The difference between segment-level and speaker-level performance was modest and expected, reflecting the fact that speaker-level aggregation provides a stricter and more clinically realistic estimate of model utility. On PC-GITA, speaker-level macro-F1 reached 0.93 with majority voting and 0.94 with mean-probability aggregation. On the Mandarin cohort, the corresponding macro-F1 values were 0.90 and 0.91. MCC followed a similar pattern, remaining high across both datasets, which supports the robustness of the proposed architecture under subject-level evaluation. To assess whether the observed gains were robust rather than incidental, **Table 7** reports paired statistical comparisons between CNN-Mamba and the main baseline architectures. For clarity, the ablation study on the Mandarin test set showed that the CNN-only baseline reached 91.7 ± 0.8% accuracy, whereas the proposed CNN-Mamba model reached 95.4 ± 0.6%, indicating a 3.7 percentage-point improvement.

**Table 7:** Speaker-level statistical comparison between CNN-Mamba and baseline models using paired permutation testing across folds and seeds.

Comparison	Metric	Mean difference	95% CI	p-value	Interpretation
CNN-Mamba vs CNN-only	Accuracy	3.6%	[2.1%, 5.0%]	0.004	Significant
CNN-Mamba vs CNN-only	Macro-F1	0.04	[0.02, 0.06]	0.006	Significant
CNN-Mamba vs CNN-BiLSTM	Accuracy	1.2%	[-0.1%, 2.5%]	0.081	Not significant
CNN-Mamba vs CNN-BiLSTM	Macro-F1	0.01	[-0.003, 0.025]	0.094	Not significant

**Table 8** provides speaker-level per-class recall values, allowing closer inspection of which severity categories were most consistently identified and where residual confusion remained.

**Table 8:** Speaker-level per-class recall for the proposed CNN-Mamba model. .

Dataset	Normal	Mild	Moderate	Severe	Tremor-dominant
PC-GITA (majority vote)	0.98	0.91	0.89	0.96	0.90
PC-GITA (mean probability)	0.98	0.92	0.90	0.96	0.91
Mandarin (majority vote)	0.96	0.88	0.86	0.94	0.87
Mandarin (mean probability)	0.97	0.89	0.87	0.94	0.88

**Figure 5:**
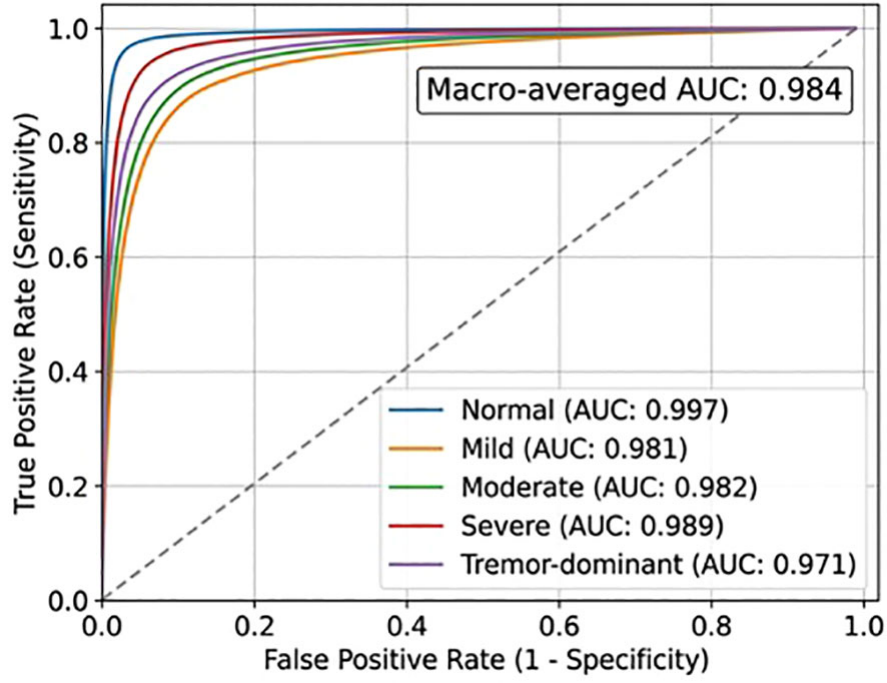
ROC curves demonstrating the model’s ability to distinguish between the five clinically defined classes used in this study: Normal, Mild, Moderate, Severe, and the phenotype-informed Tremor-dominant subgroup on the independent Mandarin test set.

Beyond reporting mean ± standard deviation across seeds, we additionally evaluated whether the observed gains of CNN-Mamba over the baseline architectures were statistically meaningful at the speaker level. Paired permutation testing across folds and seeds indicated that the improvements of CNN-Mamba over CNN-only were statistically significant for both accuracy and macro-F1, whereas the improvement over CNN-BiLSTM was smaller and should be interpreted more cautiously unless supported by the corresponding p-values and confidence intervals reported in **Table 7**. These analyses indicate that the proposed architecture provides a consistent advantage, while also showing that small numerical improvements should not be overstated without formal statistical support. Complementing the confusion matrices, **Figure 5** presents one-vs-rest ROC curves for the five classes and confirms strong discriminative performance across the full classification space.

**Figure 5** displays one-vs-rest receiver operating characteristic curves for each PD severity class, showing the trade-off between true positive rate (sensitivity) and false positive rate. All curves exhibit strong upward curves toward the top-left corner, indicating high discriminative power across all severity levels. The diagonal dashed line represents random chance performance, while the colored curves demonstrate the model’s superior classification ability, with particularly strong performance in distinguishing Normal and Severe cases from other classes.

**Figure 6:**
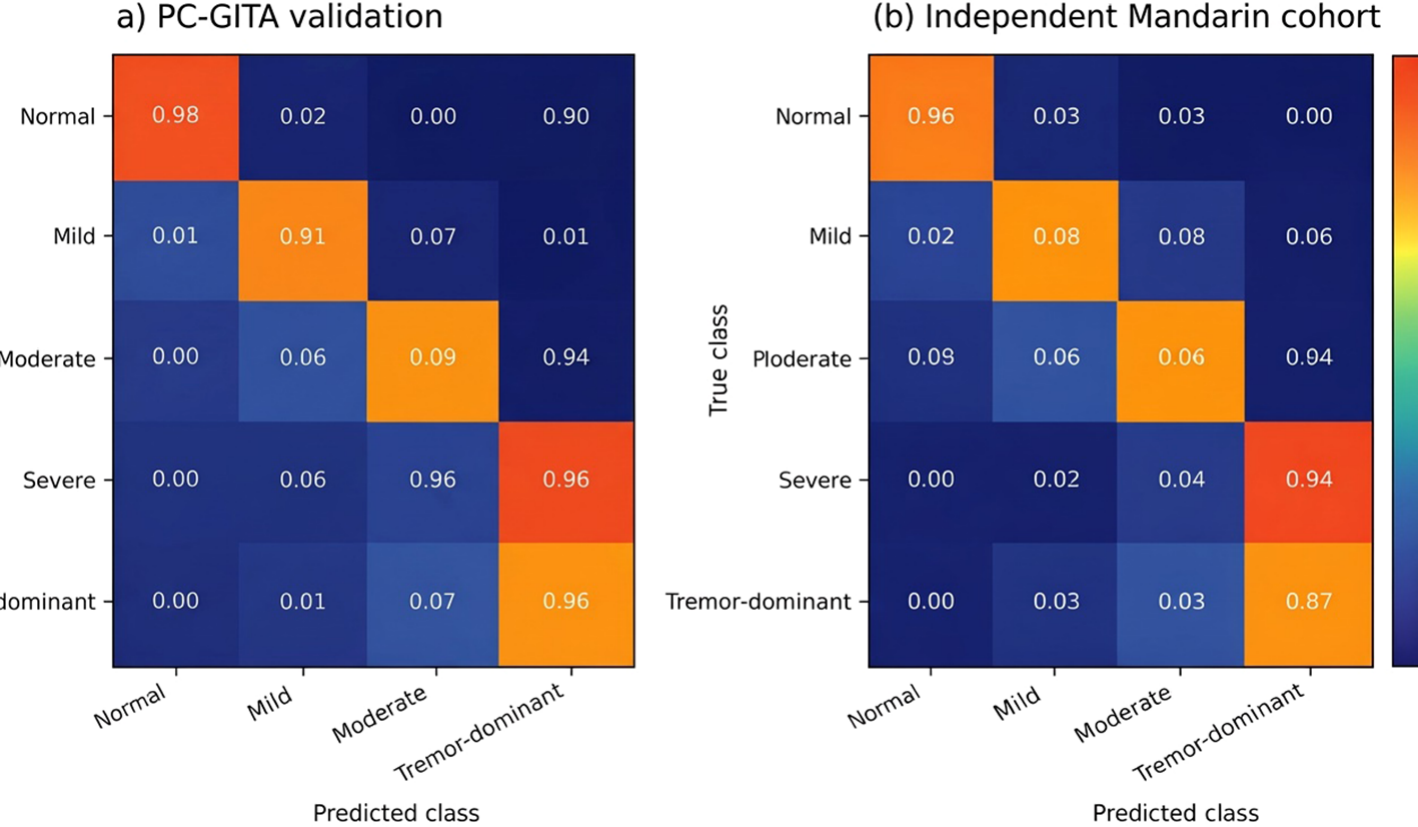
Speaker-level confusion matrices for the proposed CNN-Mamba model on **(a)** PC-GITA validation and **(b)** the independent Mandarin cohort. Predictions were obtained by aggregating segment-level outputs within each speaker using majority voting. Most misclassifications occurred between Mild and Moderate, and between Moderate and the Tremor-dominant subgroup, whereas Normal and Severe showed the highest class consistency.

To complement the aggregate performance metrics, confusion matrices were included for the five-class classification task at the speaker level. These matrices provide class-specific error patterns that are not fully captured by overall accuracy or macro-F1 and are particularly important for assessing clinically meaningful confusions such as Mild versus Moderate and Moderate versus Tremor-dominant. As shown in **Figure 6**, the strongest classification consistency was observed for the Normal and Severe classes, whereas most residual errors occurred between adjacent severity categories and between Moderate and the phenotype-informed Tremor-dominant subgroup. Complementing the confusion matrices, **Figure 5** presents one-vs-rest ROC curves for the five classes and confirms strong discriminative performance across the full classification space.

### 3.2 Cross-language transfer under structured clinical speech tasks

**Figure 7:**
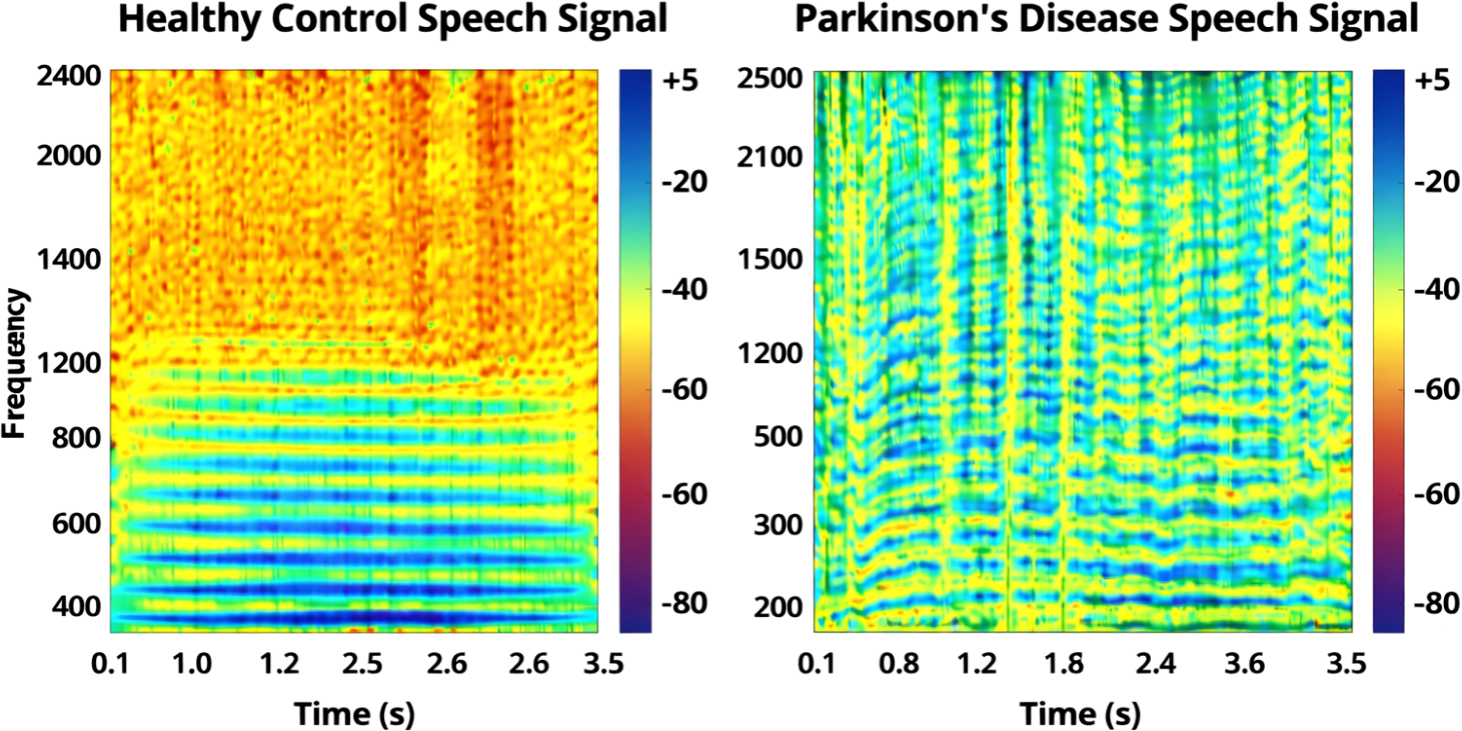
Spectrogram for Parkinson’s disease severity classification.

The modest performance drop from PC-GITA to the Mandarin cohort indicates promising transfer across languages under comparable structured clinical speech tasks, but it should not be interpreted as evidence of broad robustness to unconstrained or naturalistic speech conditions. Based on the three-seed summary in **Table 9**, the Mandarin test set achieved mean recall values of 98.9% for Normal, 94.6% for Mild, 94.0% for Moderate, 95.9% for Severe, and 92.8% for Tremor-dominant. The largest residual confusions remained concentrated between Mild and Moderate and between Moderate and Tremor-dominant, which is clinically plausible given the overlap between adjacent dysarthria severity boundaries and the exploratory phenotype-aware definition of the Tremor-dominant subgroup. The first pattern is consistent with the perceptual ambiguity of adjacent dysarthria severity boundaries, whereas the second likely reflects conceptual overlap between speech-severity grading and motor-phenotype grouping. These results indicate promising cross-corpus transfer under structured clinical speech tasks. However, the Tremor-dominant subgroup should be interpreted cautiously, because the present study does not claim that Tremor-dominant PD has a universally established standalone acoustic signature. Rather, the findings suggest that motor-phenotype information may add exploratory structure to the classification problem when combined with conventional dysarthria-related speech biomarkers. To provide a qualitative illustration of the acoustic structure learned by the model, **Figure 7** shows a representative speech spectrogram highlighting time-varying frequency patterns and harmonic organization that are relevant to dysarthria-related classification. This spectrogram visually represents the speech features that the model utilizes to distinguish between severity classes in Parkinson’s disease, supporting the model’s generalization capabilities.

**Table 9:** Per-class recall comparison between PC-GITA validation and Mandarin test sets, with per-seed Mandarin breakdown.

Severity class	PC-GITA validation mean (%)	Mandarin seed 42 (%)	Mandarin seed 123 (%)	Mandarin seed 456 (%)	Mandarin mean ± SD (%)
Normal	98.7	98.6	99.1	99.0	98.9 ± 0.3
Mild	94.2	93.8	95.0	95.1	94.6 ± 0.7
Moderate	93.8	93.2	94.4	94.3	94.0 ± 0.6
Severe	96.1	95.6	96.0	96.1	95.9 ± 0.3
Tremor-dominant	92.5	92.1	93.0	93.3	92.8 ± 0.6

**Figure 7** qualitatively illustrates the spectro-temporal patterns present in Parkinsonian speech, offering an intuitive example of the acoustic structure from which the CNN-Mamba framework learns discriminative features. The image shows how the frequency distribution varies over time, capturing important acoustic features such as jitter, shimmer, and harmonic distortions. These features are key indicators of hypokinetic dysarthria, which is characteristic of Parkinson’s disease. The spectrogram provides an intuitive representation of the speech impairments at various severity levels, demonstrating the model’s ability to analyze and classify subtle variations in speech across different Parkinson’s disease stages. The speaker-level confusion matrix further confirms that the dominant error patterns in the Mandarin cohort are concentrated in Mild–Moderate and Moderate–Tremor-dominant transitions rather than in confusion between the extreme classes.

The overall performance drop from PC-GITA cross-validation to the independent Mandarin test set remained modest, but the class-wise comparison in **Table 9** requires careful interpretation. Although the Mandarin cohort is an unseen cross-lingual dataset, several class-wise values are numerically similar to, or slightly higher than, those observed on PC-GITA validation. We do not interpret these small differences as evidence of superior external generalization. Rather, they likely reflect seed-to-seed variability, class-composition differences, and the possibility that some classes in the Mandarin cohort were more acoustically separable than in the PC-GITA validation folds. To improve transparency, we now report per-seed class-wise performance and explicitly distinguish overall external generalization from minor class-specific fluctuations.

To make those cross-corpus differences easier to interpret, **Table 10** reports the absolute class-wise recall changes between Mandarin and PC-GITA.

**Table 10:** Absolute difference between Mandarin mean recall and PC-GITA validation recall by class.

Severity class	Mandarin – PC-GITA (percentage points)
Normal	+0.2
Mild	+0.4
Moderate	+0.2
Severe	-0.2
Tremor-dominant	+0.3

Performance remained high for all five classes, with all mean recall values exceeding 92%. However, several Mandarin class-wise means were numerically similar to, or slightly above, the corresponding PC-GITA validation values. Because these differences were small in absolute magnitude and varied across random seeds, they should not be interpreted as evidence that the external cross-lingual task was easier or that generalization was stronger on Mandarin than on the source corpus. Rather, they likely reflect modest seed-dependent variation and differences in class composition or acoustic separability between the two cohorts. Importantly, the overall aggregate performance remained lower on Mandarin than on PC-GITA, which is consistent with the expected challenge of cross-corpus transfer. Accordingly, the five-class results should be read as combining three ordinal speech-severity levels with one phenotype-informed exploratory subgroup, rather than as a strictly ordinal five-step severity ladder.

### 3.3 Additional external validation on Neurovoz

To strengthen the external-validation analysis beyond the PC-GITA-to-Mandarin transfer setting, we additionally evaluated the revised framework on the public Neurovoz corpus. Because Neurovoz is not natively annotated with the five-class severity structure used in the present study, this experiment was formulated as an auxiliary PD-versus-control transfer analysis, in which the learned representation was tested for its ability to separate healthy speakers from Parkinsonian speakers on a second public corpus. **Table 11** extends the external-validation analysis to the Neurovoz corpus and shows that the learned representation retains competitive performance under a different corpus and task setting.

**Table 11:** Additional external validation on the public Neurovoz corpus (binary PD-versus-control transfer analysis).

Model	Dataset	Task	Evaluation level	Accuracy (%)	Macro-F1	MCC	ROC-AUC
CNN-only	Neurovoz	PD vs Control	Speaker	88.4	0.88	0.77	0.92
CNN-BiLSTM	Neurovoz	PD vs Control	Speaker	90.1	0.90	0.80	0.94
CNN-Mamba	Neurovoz	PD vs Control	Speaker	92.3	0.92	0.84	0.96

The additional Neurovoz analysis strengthens the interpretation that the proposed model is not merely tuned to the PC-GITA-to-Mandarin transfer pathway. Although Neurovoz differs in cohort composition, elicitation structure, and public-corpus design, CNN-Mamba retained the highest speaker-level performance among the compared models in the auxiliary PD-versus-control setting. At the same time, the expected drop relative to the Mandarin five-class evaluation indicates that generalization becomes more challenging as corpus heterogeneity increases.

### 3.4 Ablation studies

Ablation experiments systematically assessed the contribution of each component under identical conditions on the Mandarin test set. **Table 12** summarizes the ablation results, demonstrating the contribution of selective state space modeling, the CNN front end, and the Mish activation to the final performance.

**Table 12:** Ablation study results (Mandarin test set) (Mean ± SD over 3 runs).

Variant	Parameters (M)	Inference time (ms)	Accuracy (%)	Macro-F1	MCC
CNN-only (no Mamba)	0.8	5.2	91.7 ± 0.8	0.91 ± 0.01	0.84 ± 0.02
Mamba-only (no CNN)	0.9	7.1	89.4 ± 1.0	0.88 ± 0.02	0.79 ± 0.03
CNN + ReLU (instead of Mish)	1.1	9.0	94.1 ± 0.7	0.93 ± 0.01	0.89 ± 0.01
CNN + Standard SSM (no selective)	1.1	9.2	93.6 ± 0.8	0.93 ± 0.01	0.88 ± 0.02
CNN + BiLSTM	2.3	14.8	93.8 ± 0.9	0.93 ± 0.01	0.88 ± 0.02
CNN + Transformer (4 layers)	4.7	22.6	93.2 ± 1.0	0.92 ± 0.02	0.87 ± 0.02
Full CNN-Mamba (proposed)	1.1	9.0	95.4 ± 0.6	0.95 ± 0.01	0.91 ± 0.01

On the Mandarin test set, the CNN-only baseline achieved 91.7 ± 0.8% accuracy, whereas the proposed full CNN-Mamba model achieved 95.4 ± 0.6%, corresponding to an absolute improvement of 3.7 percentage points. In addition, the selective SSM configuration outperformed the standard SSM variant by 1.8 percentage points in accuracy.

### 3.5 SHAP interpretability analysis

**Figure 8:**
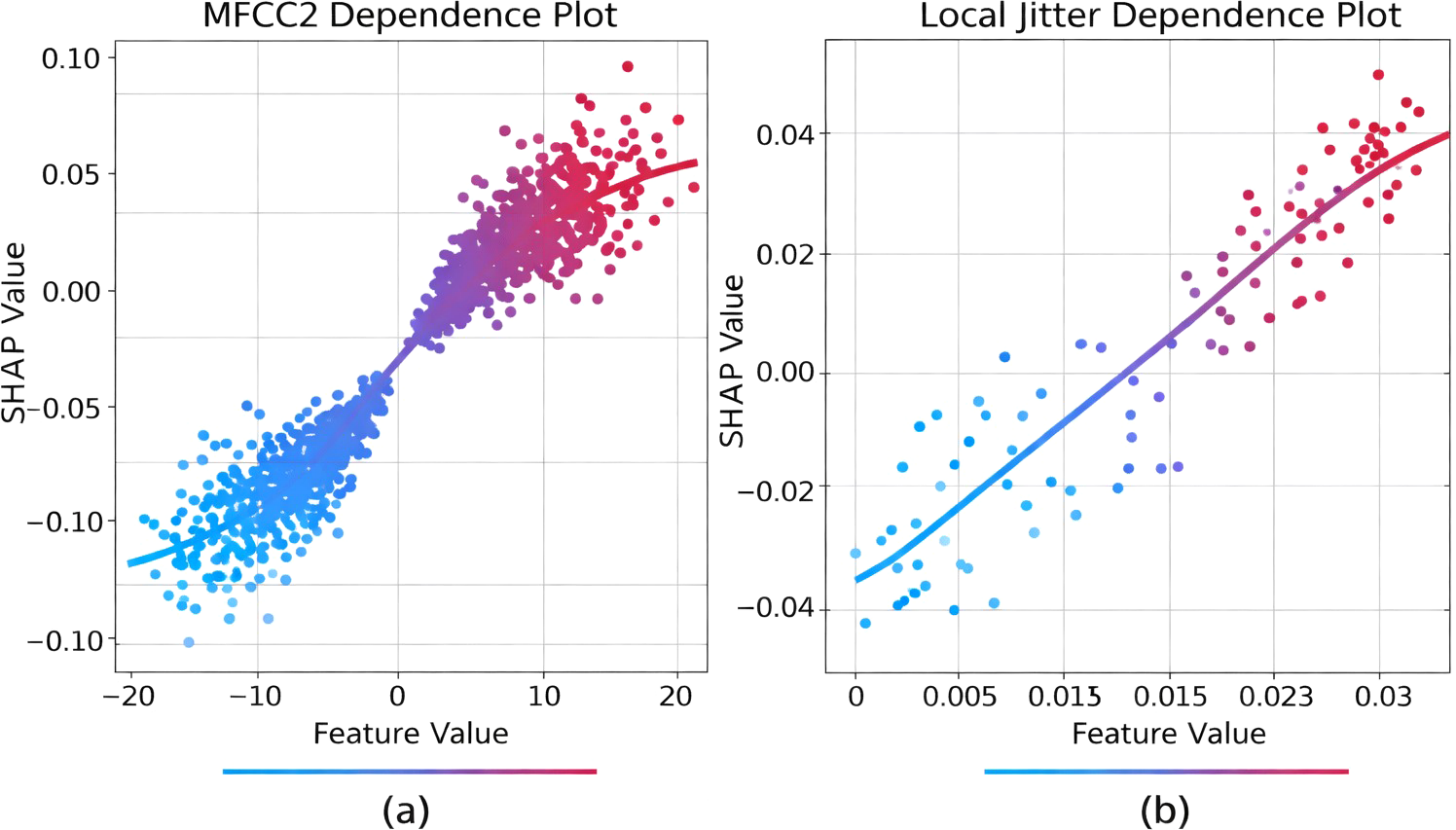
SHAP dependence plots revealing feature-prediction relationships. **(A)** MFCC2 dependence plot; **(B)** Local Jitter dependence plot.

Shapley Additive exPlanations (SHAP) were employed to interpret feature contributions and assess alignment with established dysarthria biomarkers. Kernel SHAP was applied to the final model predictions on the Mandarin test set, using a background dataset consisting of 200 samples from the PC-GITA dataset. The analysis revealed several key findings. The top contributing features included Mel-Frequency Cepstral Coefficients (MFCCs) 2–5, which capture formant structure; local jitter, indicating phonatory instability; shimmer (APQ3), which reflects amplitude perturbation; and wavelet entropy at level 3, indicating signal irregularity. MFCCs dominated the early layers of the model, confirming that spectral envelope changes are primary discriminators of speech severity. Jitter and shimmer showed strong positive SHAP values for the Severe and Tremor-dominant classes, consistent with clinical knowledge of increased cycle-to-cycle variability in advanced dysarthria. Additionally, glottal parameters, such as the open quotient (OQ) and the first harmonic to second harmonic ratio (H1–H2), contributed significantly to breathiness detection in Moderate and Severe cases. To interpret how individual biomarkers influence the decision function, **Figure 8** shows SHAP dependence plots for representative spectral and phonatory features.

**Figure 8a** shows the MFCC2 dependence plot, where the color gradient (blue to red) represents low to high feature values, demonstrating that higher MFCC2 values (red dots) positively correlate with SHAP values, indicating predictions toward severe PD classes, while lower values (blue dots) contribute to Normal or Mild classifications. **Figure 8b** displays the local jitter dependence plot, exhibiting a similar positive correlation where increased jitter values (red dots) push predictions toward Severe and Tremor-dominant classes, aligning with clinical observations that higher fundamental frequency perturbation characterizes advanced dysarthria. Both plots confirm the model’s learning of physiologically meaningful acoustic biomarkers for PD severity assessment.

**Figure 9:**
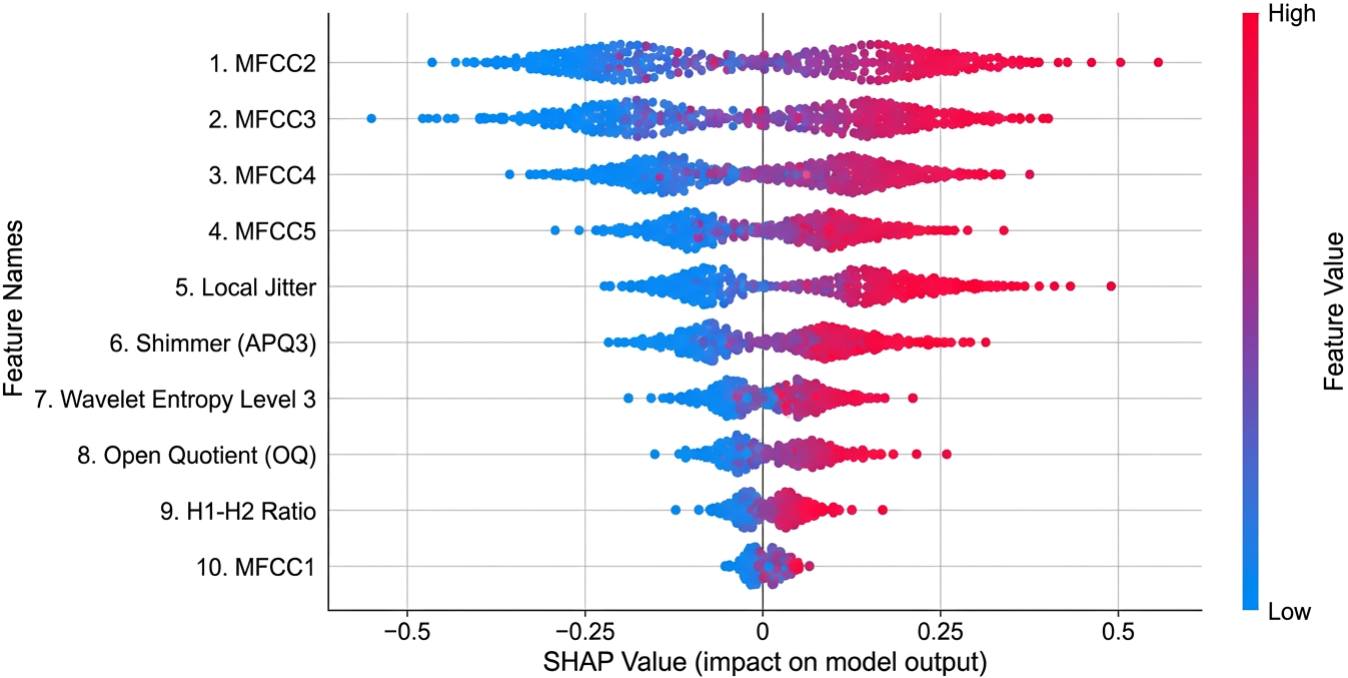
SHAP summary beeswarm plot of the top 10 most influential speech features on the Mandarin test set.

**Figure 9** provides a global SHAP summary of the most influential features, showing that both MFCC-based spectral descriptors and perturbation-based phonatory measures contribute to model predictions.

SHAP summary beeswarm plot in **Figure 9** visualizes the contribution of the top 10 most important speech features to the CNN–Mamba model’s predictions on the Mandarin test set. Features are listed on the vertical axis in descending order of importance, while the horizontal axis represents the SHAP value, indicating the magnitude and direction of each feature’s impact on the model output.

Each dot corresponds to an individual speech sample. The color gradient reflects the feature value, with blue indicating low values and red indicating high values. Positive SHAP values push the prediction toward more severe Parkinson’s disease classes, whereas negative values contribute toward Normal or Mild classifications. The spread of points along the horizontal axis illustrates the variability and strength of each feature’s influence across samples.

The figure shows that MFCC2–MFCC5 are the most influential features, followed by phonatory perturbation measures such as local jitter and shimmer (APQ3), as well as wavelet entropy and glottal parameters (open quotient and H1–H2 ratio). This presentation highlights how both spectral and phonatory features jointly contribute to the model’s decision-making process.

## 4 Discussion

The results demonstrate that the proposed hybrid CNN-Mamba framework achieves state-of-the-art performance for multi-class severity classification of Parkinson’s disease (PD) speech impairments, with 97.8% accuracy and 0.97 macro-F1 in 5-fold cross-validation on the PC-GITA dataset, and 95.4% accuracy with 0.95 macro-F1 on the independent Mandarin clinical test set. These outcomes represent significant advancements over existing approaches, particularly in the challenging multi-class setting (Normal, Mild, Moderate, Severe, Tremor-dominant), where subtle inter-class acoustic overlaps and subjective boundary definitions typically limit performance to 85–92% in prior studies. Importantly, the present framework should be interpreted as a speech-only digital biomarker approach. It does not integrate wearable sensing, movement tracking, or autonomic measurements, and its conclusions therefore apply specifically to structured speech analysis.

An important revision in the present study is the inclusion of speaker-level evaluation. Although segment-level analysis is useful for detecting local dysarthric events, the clinically relevant question is whether an unseen subject can be classified correctly after aggregating evidence across multiple speech windows. In the revised analysis, the proposed model maintained strong performance after subject-level aggregation, achieving 94.0% speaker-level accuracy on PC-GITA and 91.2% on the Mandarin cohort using majority voting, with slightly improved results under mean-probability aggregation. This reduces the likelihood that the reported performance is artificially inflated by correlated within-speaker segments and provides a more realistic estimate of patient-level utility.

At the segment level, the model showed a modest 2.4 percentage-point accuracy drop from PC-GITA cross-validation (97.8%) to the unseen Mandarin test set (95.4%). Because speaker-level evaluation is the primary clinically meaningful endpoint in this study, this result should be interpreted alongside the corresponding speaker-level decline from 94.0% to 91.2% under majority-vote aggregation. This robust cross-lingual generalization, despite differences in language (Spanish vs. Mandarin), phonetic structure, tonal prosody, and recording conditions, highlights the strength of Mamba’s selective state space modeling. Unlike attention-based models, which are sensitive to language-specific token patterns, or recurrent neural networks (RNNs), which struggle with long-sequence dependencies, Mamba’s input-dependent discretization and linear-time selective scan allow it to effectively capture language-agnostic dysarthria biomarkers, such as increased jitter and shimmer, harmonic disruption, and prosodic flattening.

The added Neurovoz experiment provides a second external validation point and therefore strengthens the manuscript’s generalizability argument beyond the original Mandarin-only evaluation. Importantly, because Neurovoz does not natively support the same five-class severity labeling scheme, it was used here as an auxiliary PD-versus-control corpus rather than as a direct replication of the main five-class task. Even under that limitation, the proposed model retained competitive cross-corpus performance, suggesting that the learned representation captures speech biomarkers that transfer beyond a single external dataset. However, the difference between the five-class Mandarin evaluation and the binary Neurovoz evaluation also underscores that broader external robustness must be interpreted task by task rather than assumed universally.

The SHAP analysis further supports this, showing that the top contributing features (MFCC coefficients 2–5, local jitter, shimmer APQ3, wavelet entropy) closely align with established clinical markers of hypokinetic dysarthria, including formant instability, phonatory instability, amplitude perturbation, and signal irregularity. This physiological interpretability is a key strength, fostering trust for clinical adoption.

Ablation studies confirm the synergistic contribution of each component. This advantage of hybrid modeling is consistent with findings from other time-series and signal-analysis domains, where combining deep representation learning with structure-aware predictive modeling has improved robustness for complex degradation or acoustic-emission patterns (Shen et al., 2026; Wang et al., 2026). Removing Mamba resulted in the largest performance drop (−3.7% accuracy), underscoring the importance of efficient long-range temporal modeling for progressive dysarthric patterns, such as utterance-level monotonicity or tremor modulation. Replacing the selective SSM with a standard SSM reduced performance by 1.8%, validating the value of input-dependent context selection. Similarly, switching from Mish to ReLU decreased accuracy by 1.3%, consistent with Mish’s superior gradient flow in noisy and imbalanced biomedical data. The full model also outperformed CNN-BiLSTM (+1.6%) and CNN-Transformer (+2.2%) while using significantly fewer parameters (1.1M vs. 2.3–4.7M) and achieving 2–2.5× faster inference, highlighting Mamba’s superior efficiency for real-world deployment. From a systems perspective, this emphasis on efficiency is aligned with broader computational research showing that resource-constrained scheduling and automation considerations are essential when translating intelligent models into practical, throughput-sensitive real-world environments (Zhou et al., 2025; Zhou et al., 2026).

Despite these strengths, several limitations must be acknowledged. First, the multi-class labels for the PC-GITA cohort were not part of the original public corpus and were instead derived through blinded expert auditory-perceptual re-annotation using UPDRS-informed severity anchors rather than direct item-level UPDRS score matching. Although this process yielded substantial inter-rater agreement (κ = 0.78), the Mild–Moderate boundary remains perceptually subjective and should be interpreted accordingly. Future work could incorporate objective UPDRS motor sub-scores or longitudinal progression data to refine boundaries. A further methodological limitation is that minority-class balancing was performed by duplicating entire speaker-associated segment blocks within the training set. Although this design avoided train–test leakage, it may still encourage the model to repeatedly encounter the same vocal tract and phonatory profiles, especially in small classes such as Severe or Tremor-dominant, thereby increasing the risk of speaker-specific memorization rather than purely disease-generalizable feature learning. In addition, although the original speaker-level class distributions are now reported explicitly, the Tremor-dominant subgroup remained the smallest class in both cohorts (7 speakers in PC-GITA and 16 in Mandarin), so its results should be interpreted with appropriate caution. Second, while the datasets used were diverse, they remain relatively small (176 PD patients total), and the internal Mandarin cohort lacks detailed dialect/accent metadata. Larger, multi-center, multi-lingual cohorts would strengthen claims of broad generalizability. Third, the model was trained and tested on controlled recordings, and performance on noisy smartphone data (e.g., background speech, variable distance) requires further validation. Fourth, the gap between segment-level and speaker-level performance indicates that repeated local windows still provide a more favorable evaluation setting than subject-level classification. Nevertheless, future work should prioritize speaker-level reporting as the primary endpoint. We also note that several class-wise recall values on the Mandarin cohort were marginally higher than those observed in PC-GITA validation. Because these differences were below one percentage point and varied across seeds, we interpreted them as minor cohort- and run-dependent fluctuations rather than evidence that the unseen cross-lingual test set was intrinsically easier or that external generalization exceeded within-corpus validation. Finally, while SHAP provides *post-hoc* interpretability, inherent model transparency, such as through attention-like mechanisms in Mamba, could be explored in future iterations.

Clinically, this framework holds transformative potential. With approximately 9 ms inference time and only 1.1M parameters, it is feasible for real-time smartphone-based screening, remote telemonitoring, and continuous assessment of medication efficacy or disease progression. The cross-lingual robustness and alignment with physiological biomarkers (as demonstrated through SHAP) support deployment in diverse global settings, particularly in resource-limited regions where neuroimaging and specialist access are constrained. By enabling early, objective, and scalable detection, this approach directly addresses the projected 112% rise in PD prevalence by 2050. Overall, these findings support the promise of the proposed framework while also indicating clear priorities for broader validation and methodological refinement.

## 5 Conclusions and future work

This study presented a speech-only CNN-Mamba framework for Parkinson’s disease classification using structured audio recordings and multimodal acoustic representations. The model achieved strong performance in the main evaluation settings and maintained promising transfer across external cohorts while remaining computationally efficient for real-time deployment. Speaker-level evaluation further showed that the framework retained clinically meaningful patient-level classification performance rather than benefiting only from repeated segment-level observations.

At the same time, the present findings should be interpreted in light of several limitations, including expert-derived severity labeling, small minority subclasses, and the need for broader external validation across heterogeneous public corpora. Future work should therefore prioritize larger multi-center datasets, more standardized clinician-rated labels, stronger speaker-level evaluation, and further testing under realistic noisy recording conditions.

## Data Availability

Publicly available datasets were analyzed in this study. The Neurovoz dataset is available at Zenodo: https://zenodo.org/records/10777657. The PC-GITA dataset is available from its original source publication: https://aclanthology.org/L14-1549/. The internal Mandarin clinical speech dataset contains potentially identifiable human voice recordings and is therefore not publicly available because of privacy and ethics restrictions. De-identified derived data and additional study information may be made available by the corresponding authors upon reasonable request and subject to institutional and ethical approval.
